# Hazard Characterization of Modified Vaccinia Virus Ankara Vector: What Are the Knowledge Gaps?

**DOI:** 10.3390/v9110318

**Published:** 2017-10-29

**Authors:** Malachy I. Okeke, Arinze S. Okoli, Diana Diaz, Collins Offor, Taiwo G. Oludotun, Morten Tryland, Thomas Bøhn, Ugo Moens

**Affiliations:** 1 Genome Editing Research Group, GenØk-Center for Biosafety, Siva Innovation Center, N-9294 Tromso, Norway; arinze.s.okoli@uit.no (A.S.O.); morten.tryland@uit.no (M.T.); thomas.bohn@uit.no (T.B.); 2 Molecular Inflammation Research Group, Institute of Medical Biology, University i Tromsø (UiT)—The Arctic University of Norway, N-9037 Tromso, Norway; diana.k.canova@uit.no (D.D.); ugo.moens@uit.no (U.M.); 3 Department of Medical and Pharmaceutical Biotechnology, IMC University of Applied Sciences Piaristengasse 1, A-3500 Krems, Austria; collinsoffor57@yahoo.com (C.O.); graceoludotun@yahoo.com (T.G.O.); 4 Artic Infection Biology, Department of Artic and Marine Biology, UIT—The Artic University of Norway, N-9037 Tromso, Norway

**Keywords:** Modified vaccinia virus Ankara, orthopoxvirus, hazard characterization, knowledge gaps, biosafety, environmental risk assessment, uncertainty analysis, smallpox, recombinant vaccines, virus vector

## Abstract

Modified vaccinia virus Ankara (MVA) is the vector of choice for human and veterinary applications due to its strong safety profile and immunogenicity in vivo. The use of MVA and MVA-vectored vaccines against human and animal diseases must comply with regulatory requirements as they pertain to environmental risk assessment, particularly the characterization of potential adverse effects to humans, animals and the environment. MVA and recombinant MVA are widely believed to pose low or negligible risk to ecosystem health. However, key aspects of MVA biology require further research in order to provide data needed to evaluate the potential risks that may occur due to the use of MVA and MVA-vectored vaccines. The purpose of this paper is to identify knowledge gaps in the biology of MVA and recombinant MVA that are of relevance to its hazard characterization and discuss ongoing and future experiments aimed at providing data necessary to fill in the knowledge gaps. In addition, we presented arguments for the inclusion of uncertainty analysis and experimental investigation of verifiable worst-case scenarios in the environmental risk assessment of MVA and recombinant MVA. These will contribute to improved risk assessment of MVA and recombinant MVA vaccines.

## 1. Introduction

### 1.1. Genetically Modified Viruses (GMVs) for Vaccination

Vaccines and vaccine vectors are termed genetically modified (GM) if recombinant gene technology is used to create the vaccine or vector. Genetically modified viruses (GMVs) or virus vectors of homologous or heterologous disease antigens are considered highly desirable for vaccinations against diseases that are difficult to treat or for which there exists no effective conventional vaccine, e.g., acquired immune deficiency syndrome (AIDS), malaria, tuberculosis and neoplastic disorders. Some of the advantages of GMVs in vaccination are the enhancement of immunogenicity without an adjuvant and induction of robust cytotoxic T lymphocyte response to eliminate virus-infected cells. Many viruses have been used as GMVs for vaccination purposes, the major ones being *Poxviridae* (Vaccinia virus; VACV), *Retroviridae* (including lentivirus), *Adenoviridae* (human Adenovirus; hAdV), *Parvoviridae* (Adeno-associated virus; AAV), *Herpesviridae* (cytomegalovirus; CMV) and *Paramyxoviridae* (sendai virus; SeV) [1,2]. Each has unique attributes and associated risks when used as a GM virus vaccine or vector.

VACV-based vectors have good historical precedence on safety in that VACV and modified vaccinia virus Ankara (MVA) were used in smallpox vaccination. VACV-based vectors were also well tolerated in clinical trials, although adverse effects were recorded in some studies at high dose (over 10^8^ pfu) of MVA [3]. Disadvantages of vectors based on VACV include limited immunogenicity and pre-existing immunity [4]. Recombinant AdV vectors have the advantage of high transduction efficiency, a high level of transgene expression, broad cell tropism and the ability to infect both dividing and non-dividing cells [5]. The AdV vectors are also well tolerated but the presence of pre-existing anti-AdV immunity is a disadvantage associated with them [6,7,8]. Similar to AdV, AAV can infect dividing and non-dividing cells and has broad cell tropism. In addition, AAV vectors also provide long-term transgene expression, however, they may require host genome integration for viral gene expression [9]. Retrovirus and AAV vectors provide long-term gene expression and they are not plagued with pre-existing immunity. However, their package size is limited to 4.5 and 7.5 kilobases respectively [10] and retroviruses are associated with various diseases, e.g., leukemia, lymphoma and AIDS [11,12]. Vectors derived from SeV show high efficiency in gene transfer and transduce both dividing and non-dividing cells [13]. SeV infects human epithelial cells efficiently and can therefore be administered intranasally. This reduces the influence of pre-existing immunity compared to intramuscular administration [13]. These advantages/benefits of the various GMVs are being exploited in improving virus-based GM vaccines, while efforts are underway to reduce their limitations.

### 1.2. Steps in Environmental Risk Assessment of GMVs

Environmental risk assessment (ERA) is governed by regulatory instruments of different countries and regions. In the European Union (EU), the ERA is governed by Directive 2001/18/EC [14] and contains the guidelines and steps (in Annex 1 part IV of the Directive). The Directive stipulates a stepwise and case-by-case process in which hazards are identified and characterized, exposure pathways evaluated and potential risks characterized. A routine ERA procedure that is based on Directive 2001/18/EC involves the following 6 steps: hazard identification, hazard characterization, assessment of likelihood of hazard and risk level, risk characterization, risk management strategies and determination of overall risk and conclusion. The aim of the ERA is to assess the risk to humans and the environment of products derived from genetic modification and to proffer management and mitigation strategies. The environmental risks associated with GMVs have been widely published [15,16,17,18,19]. In general, hazards and potential risks to the environment of GMVs are linked to shedding, survivability, the nature and toxicity of transgenes, the dissemination and transfer of novel/foreign genes to wild-type viruses as well as contact with, and infection of, non-target individuals and animals in the environment.

### 1.3. Poxviruses as Vaccine and Vaccine Vector

Poxviruses have a linear double stranded DNA genome of approximately 130 kbp (seal parapox virus)–360 kbp (canarypox virus) and their entire morphogenesis is within the cytoplasm of infected cells. A milestone in vaccinology was achieved when smallpox, a highly lethal disease caused by variola virus (VARV), a member of the genus *Orthopoxvirus* and family *Poxviridae* [20] was officially declared extinct. Variola major and minor viruses accounted for approximately 30% and 1% mortality [21]. Smallpox is believed to have emerged around 10,000 BC but the origin of VARV is unknown [22]. Some protection from smallpox was achieved by transferring material from scabs taken from smallpox patients to uninfected individuals. “Variolation,” as it was called, resulted in a 2% case fatality rate but was nevertheless introduced in Europe in the 18th century [23]. In 1796, Edward Jenner (1749–1823) introduced the principle of vaccination, demonstrating that cowpox virus (CPXV) could be used to prevent smallpox infection and this quickly gained acceptance [21]. Initially, vaccination was performed with CPXV [24,25] and later VACV became the vaccine strain of choice. The origin of VACV remains unknown and is shrouded in mystery and controversy [26]. Smallpox was estimated to be responsible for 300–500 million deaths world-wide in the 20th century alone [27] and the World Health Organization (WHO) launched a strategic plan to eliminate smallpox in 1967. Smallpox was announced eradicated at the 33rd WHO assembly on 8 May 1980 [21]. The successful eradication of smallpox was possible due to early recognition and diagnosis of the disease, quarantine of patients, effective vaccination and the fact that VARV infects only humans and has no animal host reservoir.

The discovery that VACV can be used as a vector to express heterologous transgenes in mammalian cells [28,29], the potential that VARV can be deployed as an agent of bioterrorism [30] and the increasing zoonotic potential of many orthopoxviruses (OPVs) especially VACV [31], CPXV [32] and monkeypox virus (MPXV) [33] lead to the resurgence of poxvirus research (especially vaccine development) after the eradication of smallpox. Recombinant vaccines based on poxvirus are good candidates for prophylactic and therapeutic vaccination of humans and animals against infectious diseases and neoplasms [26,34,35,36]. Poxviruses are suitable as vaccine vectors because they are thermostable, can infect different hosts and cell types, multiply only in the cytoplasm of infected cells and have capacity for insertion and expression of up to 25 kbp of foreign DNA as well as ease of scalability for commercial production. Among OPV-vectored vaccines, only Raboral V-RG (RVG), a recombinant rabies vaccines based on VACV has been registered. RGV was used to vaccinate against rabies in raccoons and foxes in North America and Europe [37,38]. Except as an oncolytic agent, multiplication competent poxvirus vaccines are not recommended for vaccination (particularly in humans) due to the risk of adverse effects [39,40]. Multiplication incompetent poxvirus vectors have been developed as a safer alternative to multiplication competent vectors and these include MVA, attenuated VACV Copenhagen (NYVAC) and attenuated derivatives of fowlpox virus (TROVAC) and canarypox virus (ALVAC). These multiplication deficient poxvirus vectors form the backbone of most poxvirus-vectored vaccines that are in preclinical development, clinical trials or under marketing authorization application (MAA) evaluation [26,41].

### 1.4. MVA Is a Safe Vector—Need for Hazard Characterization?

MVA is arguably the most promising virus vector due to its capacity to express a wide array of transgenes with correct post-translational modification and its immunogenicity in vivo along with its safety profile including host restriction in mammalian cells and attenuation in vivo. Both domestic and international legislations require that the ERA of virus-vectored vaccines be submitted and evaluated as part of MAA and clinical trial application. Of particular interest to the ERA is the characteristics of the MVA vector, its interaction with the host and the environment that may result in immediate or delayed risk to human and animal health as well as to the environment. The strong safety profile of MVA-vectored vaccines as well as its history of safe use as smallpox vaccine have stagnated research into aspects of the biology of the vector that are still poorly understood but also relevant for further optimization of the safety of MVA. Thus, there is an underlying assumption that MVA is so safe that studies aimed at risk characterization of the vector is either regarded as irrelevant or has been unequivocally addressed in scientific literature [42].

Hazard characterization (ERA step 2) of MVA and MVA-vectored vaccines would require knowledge of: (i) nature and distribution of naturally circulating OPVs that may rescue the MVA during co-infection and superinfection; (ii) recombination between MVA-vectored vaccines and naturally occurring OPVs during co-infection/superinfection of cell cultures, immune-competent and immune-compromised animals; (iii) MVA host restriction in human cells and the molecular basis for the restriction; (iv) clonal purity of MVA stocks; (v) genome and transgene stability of MVA and MVA-vectored vaccines following multiple serial passages; (vi) virus and host factors that modulate transgene stability; (vii) effect of the expressed transgene(s) on the virus-host transcriptome, proteome, metabolome and epigenome and (viii) biodistribution and shedding in immune-competent and immune-compromised individuals.

The aim of this review is to identify gaps in knowledge with respect to hazard characterization of MVA vector/MVA-vectored vaccines and highlight ongoing and future experiments aimed to address these knowledge gaps. This will contribute to a more robust ERA of MVA vector/MVA-vectored vaccines and further optimization of MVA as a safe vaccine vector.

## 2. Modified Vaccinia Virus Ankara

### 2.1. Origin and History of Use as a Smallpox Vaccine

Different variants of VACV were used for vaccination against smallpox. MVA was originally generated by serial passages of chorioallantois Vaccinia virus Ankara (CVA) in chicken embryo fibroblast cells (CEF). It was produced in the Bavarian State Vaccine Institute (Germany) during 1968–1985 and used as a human smallpox vaccine [43]. The serial passages (>570 times) of the virus in CEF cultures resulted in major deletions and mutations in the viral genome, with a loss of approximately 30 kbp [44]. These modifications also affected virulence and immune evasion functions [45,46,47], resulting in a highly attenuated vaccine virus that produced a low degree of adverse effects on the vaccinees [48]. A complete replication cycle of MVA is restricted to avian cells. In human and other mammalian cells, the virus replicates its DNA, has unimpaired expression of early and intermediate genes as well as most late genes but has a block at the stage of virion assembly [34] and thus cannot produce infectious progeny [49,50,51,52]. However, MVA is still able to express viral genes, including inserted genes (i.e., encoding antigens to which the vaccine is directed) and thus stimulate the immune system of the recipient in a relevant way.

The MVA vaccine against smallpox was administered mostly intradermally or subcutaneously. It was often used in Germany from 1968 to 1988 as a first vaccination, usually to vaccinia-naive children and adults that could be at risk for adverse effects if vaccinated with other and less attenuated smallpox vaccines [53]. The MVA vaccine elicited a weak virus neutralizing antibody response, as a first priming of the immune system, which was followed by a VACV smallpox vaccination resulting in an effective immune response and protection against smallpox. However, it was later discovered that the weak neutralizing antibody elicited by MVA priming is because the hemagglutinin (HA) protein to which VACV antibody neutralizing assay is based on is absent in MVA. This is due to the truncation of the HA promoter [46]. As a response to MVA, vaccinees experienced mild local reactions such as reddening on the vaccine inoculation site, but did not develop blisters, pustules or ulcers. Of 7098 vaccinees, 2.3% developed fever (i.e., >38 °C) and 4.1% developed non-specific general symptoms [53]. The MVA vaccine was licensed in Germany in 1977 and was administered to over 120,000 persons. However, these vaccinations were conducted when smallpox was no longer endemic in Germany and thus its immunological efficacy against a fully virulent VARV remains unknown [54], but it has proven to protect primates from lethal monkeypox infection [55].

The development and optimization of MVA as a smallpox vaccine continued post smallpox eradication. This in part is driven by the concern that the smallpox virus may re-emerge, either due to accidental escape from containment or due to bioterrorism. Hence, there is a public health need to optimize MVA as an effective but safer alternative to multiplication competent smallpox vaccines. The European Medicines Agency (EMA) has registered Imvanex^®^ (MVA-BN), as a smallpox vaccine. MVA-BN is a single clone derived from MVA-584 following six rounds of plaque purification [56]. Currently, MVA-BN is the most host restricted MVA variant in vitro as well as the most attenuated in vivo [56].

### 2.2. Major Characteristics of MVA

MVA was developed from CVA by over 570 passages in CEF. This resulted in the loss of about 15% (approximately 30 kbp) of the parental CVA genome [45,46] and several genes coding for host range factors and immunomodulators are either lost or fragmented [46,57]. MVA is highly host restricted and undergoes abortive infection in all human and other mammalian cells tested so far, except in Baby Hamster Kidney (BHK-21) cells [51], rat small intestinal epithelial cells 6 (IEC6) [52] and cell lines derived from Egyptian fruit bat [58]. However, there is a significant variability in the susceptibility of MVA strains to mammalian cell lines (Table 1). MVA-1721 productively infects some human cell lines while MVA-BN undergoes abortive infection in mammalian cell lines (Table 1). Even in some non-permissive cells as determined by fold increase in virus titer [50], there is limited production of mature MVA virions [52].

The attenuation of MVA in vivo depends on the strain or variant. Most MVA strains produce abortive infection in intact animal hosts and have been shown to be non-pathogenic even in immune-deficient animals and humans [59,60,61,62], although some variants were virulent to immune-deficient mice [56]. Compared to multiplication competent VACVs, MVA induces a more rapid immune responses and activation of the innate immune system [63].

### 2.3. MVA as a Vaccine Vector

Following the discovery in 1982 that VACV can express heterologous genes efficiently in mammalian cells [28,29], MVA has undergone rapid development as a virus vector in vaccines against infectious diseases and cancers. The unique characteristics of non-productive infection in human cells [49], attenuation in vivo [56], generation of rapid immune responses [63] and the robust activation of the innate immune system [64] have positioned MVA as the virus vector of choice. Although, no vaccine has been licensed for human use, several MVA-vectored vaccines against HIV/AIDS [65,66,67], tuberculosis [68], malaria [69,70,71], Ebola [72,73], influenza [74,75] and hepatitis B [76] are already in clinical trials, while several candidate vaccines against a plethora of other human diseases are in pre-clinical trials [26,34,77]. Apart from human infectious diseases, recombinant MVA vaccines against cancers are in pre-clinical and clinical development [78,79,80,81]. MVA is also an attractive and efficient virus vector for the development of recombinant vaccines against diseases of domesticated animals and wildlife [82,83,84,85,86]. Although MVA has shown great promise as a vector for recombinant vaccines, there is still the need to improve its efficacy and safety. MVA is further optimized for improved vaccine efficacy by deleting the few intact VACV immunomodulatory genes [87,88,89], insertion of genes that encodes co-stimulatory molecules [90,91] and more precise regulation of gene expression from endogenous or synthetic promoters [92,93,94]. On the contrary, studies aimed at further attenuation of the MVA vector are miniscule probably due to the assumption that the development of MVA has reached a satisfactory level of safety and that additional attenuation may compromise its immunogenicity.

### 2.4. Priorities for MVA Research: Is Environmental Risk Assessment a Priority?

The MVA vector is generally believed to pose a very low or negligible risk to humans, animals and the environment [17,19,42]. It is claimed that there is overwhelming and indisputable scientific evidence [42] to support this assertion. However, such scientific consensus will be undermined if research on the biosafety and risk characterization of the MVA vector is omitted, not prioritized or remains unpublished. The number of peer reviewed articles published on the two arms of MVA vaccine research being: (i) efficacy (immunogenicity) and (ii) biosafety (environmental risk assessment), was chosen as an indicator of research priority. The object of the PubMed search is “Modified vaccinia virus Ankara” and keyword(s) were appended to the object as prefix or suffix. Each pre-fixed or suffixed object was searched independently and the number of published articles was recorded. PubMed search was conducted on 13 July 2017. When queried with the object “Modified vaccinia virus Ankara” and “Modified vaccinia virus Ankara vaccine” 866 and 800 articles respectively were retrieved. Keywords dealing with the efficacy or immunogenicity of MVA returned higher numbers of published articles (223 to 478), whereas keywords dealing with biosafety or ERA returned very few hits (3 to 16). When the database was queried with the keywords “MVA safety” instead of “MVA biosafety,” the number of articles increased from 16 to 183. However, safety studies are not biosafety or environmental risk studies as they are often misconstrued to be. Safety studies deal with potential adverse effects on the patient (direct recipient of the MVA vaccine), whereas ERA studies deal with the evaluation of potential adverse effects to the environment outside of the patient [19]. Since the generic keywords dealing with ERA or biosafety generated very few hits, we expanded the search by using several specific keywords like “MVA off-target effects,” “MVA genome stability,” “MVA shedding,” “MVA biodistribution” and others. Again, they returned a very low number of published articles (0 to 40) with the exception of “Modified vaccinia virus Ankara recombination” which returned 93 hits. However, except for three articles dealing with recombination in a risk assessment context [19,95,96], the remainder deal with the construction of recombinant MVA. The PubMed search demonstrated that there is an enormous data pool on the immunogenicity of MVA but little peer-reviewed data on its biosafety or ERA.

## 3. Knowledge Gaps and Omitted Research

### 3.1. Host Cell Restriction of MVA in Human and Other Mammalian Cells

The inability of MVA to undergo productive infection in human and most cells of mammalian origin is the main reason why MVA is regarded as a safe virus vector. In approving the licensing of Imvanex^®^ as a smallpox vaccine within EU, the EMA Committee for Medicinal Products for Human Use (CHMP) stated, “With regard to safety, the vaccinia virus in Imvanex cannot replicate in human cells and hence is less likely to cause side effects than previous smallpox vaccines. Imvanex would therefore be beneficial for people who cannot be given vaccines containing replicating viruses such as patients with weakened immune system” [97]. The molecular basis for the non-productive infection of human and other mammalian cells by MVA remains unknown. Thus, this creates a major knowledge gap in the hazard characterization of MVA. Until the molecular determinant of the host restriction is identified, it is not a certainty that MVA cannot multiply efficiently in human cells. Earlier suggestions that the absence of host range genes and the presence of six major deletions in the MVA genome may be responsible for the severe host cell defect have been shown to be incorrect as: (i) most of the replication competent VACV host range genes are still intact in MVA [46,98]; (ii) marker rescue of non-functional host range genes (*KIL* and *C7L*) with functional copies did not restore the host cell tropism of wild-type CVA [45,98]; (iii) marker rescue with large fragments of the CVA genome did not restore the wild-type phenotype [99] and (iv) most importantly the sequential introduction of the six major deletions into the CVA genome did not re-create the MVA host restricted phenotype [47]. Thus, the six major deletions and the absence of two VACV host range genes are not essential for the severe host range defect of MVA.

**Figure 1:**
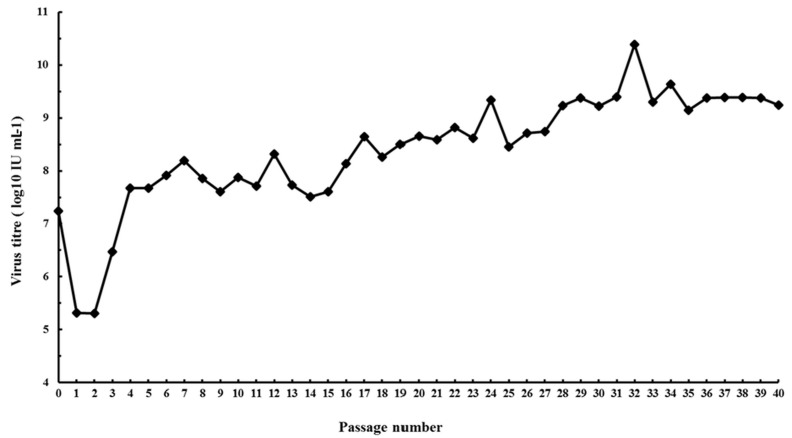
Serial passage of Modified vaccinia virus Ankara (MVA) (ATCC VR-1508) in human cell line. Caco-2 (ATCC HTB-37) cells were infected with purified MVA at a multiplicity of infection (m.o.i.) of 0.01 and the virus was blindly passaged in Caco-2 40 times. At each passage (three days post infection) infected cells were harvested, freeze-thawed three times and the virus titer was determined [52].

**Figure 2:**
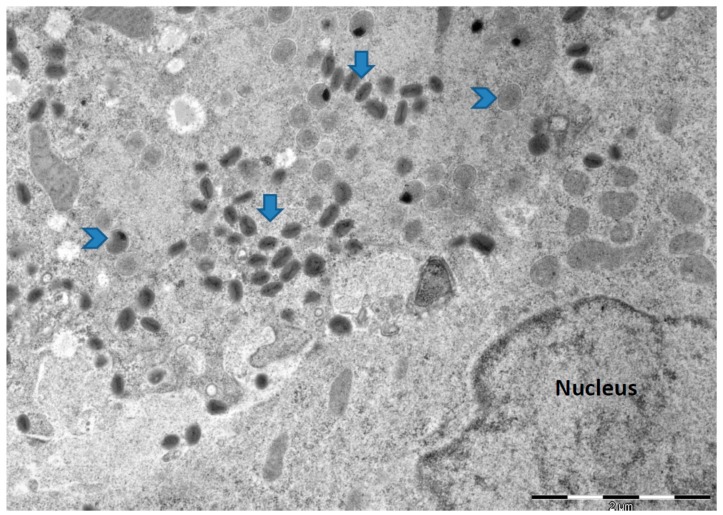
Electron micrograph of cell section of Caco-2 (ATCC HTB-37) cells infected with MVA obtained from passage 32 (p32). The infected cells were fixed 24 h post infection and processed for electron microcopy. Arrows indicate mature virions while arrow heads point to immature viruses. Similar electron micrographs were obtained for viruses harvested from p28 to p40. No mature virions were observed from cells infected with non-passaged MVA. Bar: 2 µm.

Research on identifying the molecular determinants of the MVA host cell defect in human and other mammalian cells have stagnated presumably because of the belief/assumptions that MVA cannot undergo productive infection in human cells. At present, the role of insertions/deletions (indels) and single nucleotide polymorphism (SNP) in MVA non-productive infection of human cells either singly or in combination with the six major deletions remain uninvestigated. There is also no attempt to re-adapt MVA to multiply in human cells as a way of understanding the virus and host cell determinants of host cell tropism. Since MVA host cell restriction in human and other mammalian cells was achieved by multiple serial passage in CEF, we hypothesized that MVA can be re-adapted to multiply in human cells by multiple serial passaging in human cell lines. We infected non-permissive human Caco-2 (ATCC HTB-37) cells [52] with MVA (ATCC VR-1508) at a low multiplicity of infection (m.o.i.) of 0.01 and blindly passaged it for 40 times. We were able to show 2 to 3 log fold increase in virus titer from p28 to p40 (Figure 1) and production of mature virions (Figure 2). We were able to isolate plaque purified MVA variants from p28 to p40 that were able to undergo efficient productive infection in Caco-2 cells. These observations demonstrate that at least in Caco-2 cells, the host defect of MVA may be reversible. Alternatively, it may indicate that in spite of over 572 passages and extensive plaque purification, variants capable of productive infection are still present within the MVA vaccine stock (ATCC VR-1508). Currently, we are investigating the productive infection potential of these MVA variants in other human cell lines and primary human cells as well as sequencing their genomes and comparing it to the non-passaged MVA (ATCC VR 1508). Future work will extend these experiments with MVA-VR1508 to MVA-BN and examine the attenuation/virulence potential of these Caco-2 productive MVA variants in immunocompetent and immunocompromised in vivo infection models. The results from these studies will shed light to the host restriction defect of MVA in human cells and will be of relevance to the ERA of the MVA vector if the results of the in vitro studies are replicated in vivo.

### 3.2. Clonal Purity and Genome Stability of MVA

A robust hazard characterization of a virus vector will demonstrate that the vector is clonally pure and genetically stable at a passage level and titer that is equivalent to what is present in the production batch. The MVA vector is assumed to be homogenous following over 570 passages in CEF and subsequent three rounds of plaque purification [100]. The identical genome sequence of independent MVA strains from at least five independent laboratories is presented as evidence of MVA clonal purity and genome stability [42]. However, the next generation sequencing approach used in sequencing those genomes only reported the genome of the predominant MVA clone or variant, since it was not designed to determine if minor variants are present. A demonstration of the clonal purity of MVA will require deep sequencing of the stock or master seed virus (MSV) in tandem with sequencing of several MVA clones obtained from the stock following at least three rounds of plaque purification. While Dryvax^®^ vaccine has been shown to be polyclonal using genome wide sequencing designed to examine if variants are present [101], isolation and genome wide mapping of variants within the MVA stock or MSV have never been reported. One study employing passage of MVA strains in immune-deficient mice has shown that the examined MVA strains with the exception of MVA-BN are polyclonal [56]. However, the observation from that study showing MVA-BN to be homogenous can only be confirmed if MVA-BN is subjected to deep sequencing in combination with genome sequencing of independent clones isolated from MVA-BN.

Since MVA is subjected to selection pressure (multiple multiplication cycles) during production of a high titer virus stock intended for vaccination, it is essential to examine the genome stability of the virus across MSV + 1 to MSV + 5 in human cell lines and in immune-competent and immune-compromised animal models. At present, this information is not provided in ERA dossiers submitted for clinical trials or MAA because relevant EU directive (Directive 2001/18/EC) has not made the provision of such data obligatory. Data on evolution of MVA in permissive cells during Good Manufacturing Practice (GMP) manufacturing will enable risk assessors to evaluate the genome stability of MVA and the presence of variants in the production batch intended for vaccination. The emergence of quasi-species and their role in the modulation of the MVA phenotype including host range, infectivity, attenuation, virulence and biodistribution is an omitted research field that needs to be addressed.

### 3.3. Transgene and Genome Stability of MVA-Vectored Vaccine

In order to reach the scale required for current GMP of MVA-vectored vaccines intended for clinical application, the vaccine needs to be amplified multiple times. Such high titer amplification creates a selective pressure that may result in instability of the recombinant MVA. Instability may occur within the transgene expression cassette (transgene instability) or outside the expression cassette (genome instability). Provision of genome sequences of MSV for up to five passages is necessary in order to evaluate the genome stability of recombinant vaccines based on MVA. However, provision of whole genome sequences is not mandatory under Directive 2001/18/EC. Thus, the genome stability of all MVA-vectored vaccines intended for clinical application remains unknown and potential mutations outside the expression cassette and the phenotypic implications of such mutations go unreported. Transgene stability of recombinant MVA-vectored vaccine is routinely examined during hazard characterization. In 2004, our group reported that the influenza virus hemagglutinin (HA) transgene in some CPXV-MVA recombinants is unstable resulting in the loss of the transgene following serial passages [95] and southern blot analysis suggested that the HA transgene in the first recombinant MVA-vectored vaccine is unstable [95]. Other groups have also reported transgene instability in some MVA-vectored vaccines [102,103,104]. Transgene stability is essential for robust ERA since the transgene is the logical tag for tracking the spread of the vaccine virus from target recipients to non-target species. Consequently, the loss of the transgene may preclude the monitoring of non-target effects from released or escaped recombinant MVA vaccines.

Studies on viral and host factors that modulate transgene stability are miniscule. With few exceptions, the effect of transgene insertion site [105,106,107,108], promoter choice [104], promoter spacer length [109], expression levels of the transgene [110], timing of transgene expression [104], sequence/structure of the transgene/flanking region [111] and host cell used for virus amplification [96] on transgene stability are poorly understood. Elucidating the virus and host determinants of transgene instability is essential as such knowledge will be deployed in designing transgenic MVA vaccines where the probability of transgene instability is extremely low or negligible. The few studies on transgene stability were done in established cell lines. To our knowledge, there is no report where transgene instability and the underlying mechanisms have been investigated in vivo. This is particularly relevant in immuno-compromised infection models. Immuno-compromised humans, domesticated animals and wildlife are population subsets that may be exposed to recombinant MVA vaccines, thus the stability of the transgene in immuno-compromised animal models should be investigated and data obtained should guide the ERA of recombinant MVA.

### 3.4. Geographic Distribution and Occurrence of Naturally Circulating Orthopoxviruses

The interaction between virus-vectored vaccines and naturally circulating wild-type viruses is essential to ERA since baseline data on the occurrence, nature and geographic distribution of the later is required in order to evaluate the potential for recombination, complementation, reactivation or reversion to wild-type virus. In case of MVA, this will require knowledge of the occurrence, distribution and characteristics of naturally circulating OPVs in locations and ecosystems in which recombinant MVA vaccines are to be deployed, especially for the vaccination of domesticated animals and wildlife. With the exception of Germany [112,113,114,115,116], United Kingdom [115,117,118], Fennoscandia [119,120,121,122,123,124], USA [125,126,127,128], Brazil [129,130,131,132] and India [133,134,135], data on the occurrence and characteristics of wild-type OPVs in remaining regions of the globe are limited or non-existent. MVA-vectored vaccines against malaria [136,137], HIV/AIDS [138], tuberculosis [68], Middle East respiratory syndrome coronavirus (MERS-CoV) [83], Ebola [73] and several other human and animal diseases [26,86] are in trials in Africa, Asia and Middle East, but knowledge of the characteristics and reservoir animal species for wild-type OPVs in these regions are largely non-existent. Consequently, no scientifically valid inference can be made on the potential of virus-virus interactions between recombinant MVA vaccines and naturally occurring virus relatives in these regions.

Even when recombinant poxvirus vaccines are released in areas where some knowledge exists on the characteristics and occurrence of naturally circulating relatives, no systematic study has been undertaken post release to monitor the occurrence of potential adverse effect due to the interactions between genetically modified poxvirus vaccines and naturally circulating OPVs. Raboral-VRG, a VACV-vectored rabies vaccine [139] was, alongside the attenuated rabies virus vaccine, successfully used in some regions of Belgium and France during the eradication of fox rabies in western parts of Europe in a campaign that started about three decades ago. Surveillance programs to monitor the efficacy of the vaccination (i.e., prevalence of rabies) have been ongoing [140], but no report exists on the interaction between VRG and naturally circulating OPVs in foxes and other wildlife in Europe. A comprehensive, genome wide mapping of OPVs in foxes and other animals in the foxes’ food chain in the regions where the Raboral-VRG bait vaccine has been used would yield data that would help risk assessors evaluate the potential for recombination, complementation and reversion of the vector back to wild type. Thus, the absence of data on naturally circulating OPVs before and after release of recombinant poxvirus (including MVA) vectored vaccines is a significant knowledge gap in the ERA of MVA-vectored vaccines.

### 3.5. Recombination between MVA-Vectored Vaccine and Other Orthopoxviruses

Recombination between poxvirus-vectored vaccines and naturally circulating OPVs during co-infection and superinfection of cells/hosts is significant in terms of ERA. This is because recombination has the potential of restoring productive infection of a multiplication-incompetent vaccine-vector as well as generating hybrid viruses with altered host range, cell tropism, transmissibility and virulence. Although recombination is known to occur among replicating poxviruses [141,142,143,144] and is indeed the method for constructing recombinant poxvirus-vectored vaccines [145], studies aimed at investigating recombination between MVA-vectored vaccines and other OPVs are rare. Previously, we have demonstrated that recombination occurred between an MVA-vectored influenza vaccine and naturally circulating CPXV during co-infection of BHK-21 cells [95] and generated progeny hybrid viruses with parental and non-parental characteristics [95,96]. Provision of data on the potential for recombination between the MVA-vectored vaccine and wild-type OPVs is not obligatory under Directive 2001/18/EC [14], which may explain the paucity of data on this issue. At present, the ERA of MVA-vectored vaccine does not evaluate the potential for recombination between the recombinant MVA vaccine and naturally circulating OPVs because the risk of recombination is said to be negligible [19]. Recombination between recombinant MVA vaccines and wild type OPVs is deemed unlikely because MVA does not produce infectious virions in human and most other cells of mammalian origin [50,52,99] as well as due to repulsion of superinfecting virions [146,147,148]. However, abortive infection and repulsion of superinfecting virions are not sufficient to prevent recombination since recombination requires the presence of homologous DNA only and repulsion of infectious virions is not absolute but leaky [149]. Although, OPV infections are short-lived, DNAemia lasting for up to four weeks has been reported for CPXVs [150] and in this time window, recombination can occur if MVA or recombinant MVA is used to vaccinate such a host. Since DNA replication is not impaired in MVA during non-productive infection of semi- and non-permissive cells, recombination between MVA and wild-type OPVs during co-infection and superinfection cannot be excluded.

**Figure 3:**
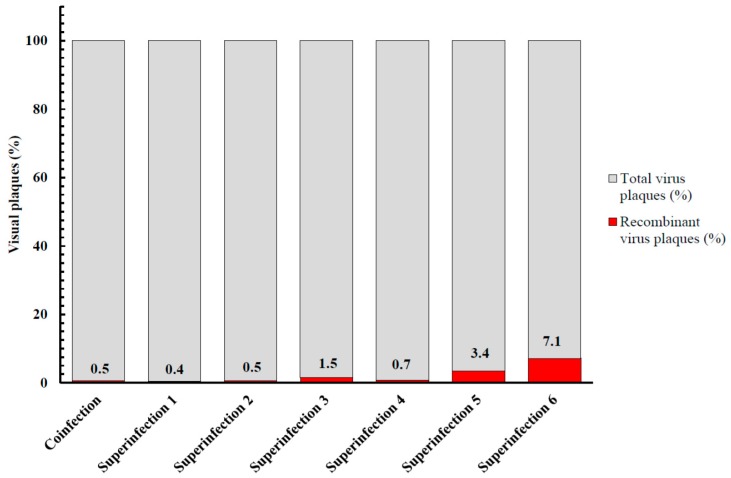
Total and recombinant virus yield in Vero cells. Co-infection, simultaneous infection of cells with Modified vaccinia virus Ankara-vectored influenza vaccine (MVA-HANP) and feline cowpox virus (fCPXV); Superinfection 1, primary infection with MVA-HANP and superinfection with fCPXV at two h post primary virus infection (ppi); Superinfection 2, primary infection with fCPXV and superinfection with MVA-HANP at two h ppi; Superinfection 3, primary infection with MVA-HANP and superinfection with fCPXV at four h ppi; Superinfection 4, primary infection with fCPXV and superinfection with MVA-HANP at four h ppi; Superinfection 5, primary infection with MVA-HANP and superinfection with fCPXV at six h ppi and Superinfection 6, primary infection with fCPXV and superinfection with MVA-HANP at six h ppi. Co-infection and superinfection were done at a m.o.i. of 5.0 for each virus and cultures were harvested three days ppi.

**Figure 4:**
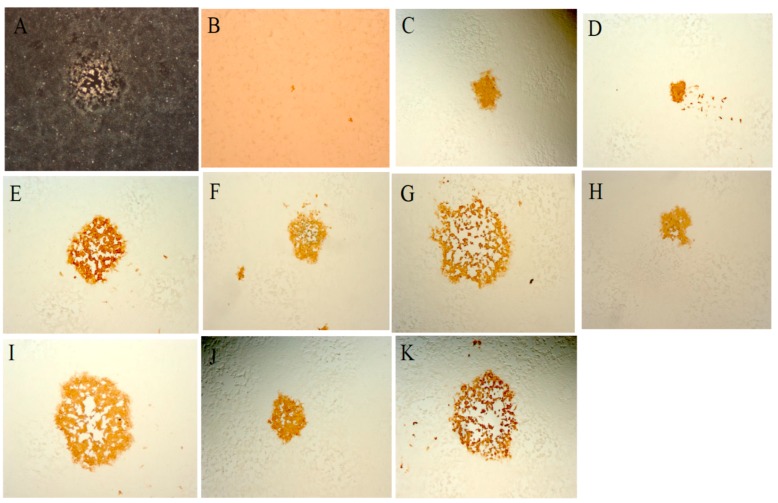
Plaque formation by hybrid progeny viruses in co-infected and superinfected Vero cells. Cells were co-infected with the parental viruses at a m.o.i. of 5.0 for each virus. Cells were harvested three days post infection, freeze-thawed and sonicated. The harvested cell suspension was used to inoculate fresh monolayer of Vero cells. Recombinant viruses expressing the influenza virus hemagglutinin (HA) transgene were detected by standard immunostaining. For superinfection 1, Vero cells were infected first with MVA-HANP, adsorption was for an h. Medium was removed and cells were washed twice with cold PBS and then superinfected with fCPXV at two h post primary virus infection (ppi). In superinfection 2, cells were first infected with fCPXV and superinfected with MVA-HANP at two h ppi. (**A**) Vero cells infected with parental strain fCPXV; (**B**) Vero cells infected with parental strain MVA-HANP; (**C**–**E**), Representative panel of Vero cells co-infected with parental strains; (**F**–**H**) Panel of Vero cells in superinfection 1; and (**I**–**K**) Panel of Vero cells in superinfection 2. All panels show representative fields at approximately 200× magnification.

To examine the potential for recombination in cells in which MVA multiplies poorly, Vero cells were co-infected with MVA-vectored influenza vaccine (MVA-HANP) [151] and a feline CPXV (fCPXV) [119], infected with MVA-HANP or fCPXV respectively and thereafter superinfected with either fCPXV or MVA-HANP at various times post primary virus infection (ppi). Our results showed that recombination occurred and that 0.5% of the visual plaques in co-infected cells and 0.4% to 7.1% of plaques in superinfected cells expressed the transgene (Figure 3). Both the transgene expressing MVA/fCPXV recombinant progenies (Figure 4) and non-transgene expressing MVA/fCPXV recombinants displayed different plaque phenotypes.

These results demonstrated that: (i) recombinant MVA vaccine can recombine with naturally circulating OPVs during co-infection and superinfection of mammalian cells that are semi-permissive to MVA and (ii) repulsion of superinfecting virions does not prevent recombination between recombinant MVA and wild-type OPVs. At present, we are extending these experiments to non-permissive cells and to immune-competent and immune-deficient animal models, as well as mapping genome-wide patterns of recombination in the already isolated recombinants. If our results from cell cultures are replicated in intact animal models, the potential for recombination should be examined for each MVA-vectored vaccine for which MAA and clinical trial application is being sought. In addition, the present emphasis on the likelihood or frequency of occurrence but not on the consequences needs a re-examination. Consideration should also be given to the impact or consequences of a presumably rare event like recombination. A rare recombination event may generate progeny hybrid viruses that are more virulent than any of the parental strains. Naturally occurring OPVs like CPXV, MPXV or VACV may become more virulent if they acquire the transgene encoding co-stimulatory molecules from an MVA-vectored vaccine following an unlikely recombination event. Among alphaherpesviruses, recombination between attenuated infectious laryngotracheitis virus vaccine strains resulted in the emergence of virulent field isolates [152].

### 3.6. Biodistribution, Shedding and Persistence of GMVs

A vaccine virus/vector will be dispersed to cells, tissues and organs of the patient (biodistribution) from the initial administration site (e.g., injection site). The virus can be introduced into the environment via body fluids (e.g., saliva, sweat, urine, feces, nasopharyngeal fluids, blood, exudates from skin lesions, breast milk and semen of the vaccinee (shedding). Following shedding, survival of the shed virus/vector outside the host (persistence) will depend on the virus/vector biological properties and the nature of the environment. Thus, assessment of pathways through which genetically modified and/or transgenes may interact with the ecosystem (other than the vaccine recipient) is critical in ERA. In this Section, the relevance of biodistribution, shedding and persistence of GMVs to ERA is briefly discussed.

#### 3.6.1. Biodistribution

The virus/vector/transgene can potentially be dispersed throughout the body when administered via a route other than the alimentary canal and this has potential of transmission to sex cells (with the attendant risk of transmission to offspring). MVA can reach target tissues other than the site of administration [153,154]. However, being multiplication incompetent, MVA is rapidly cleared from the tissues [155,156]. In situations where genetic modification has altered tissue tropism of the GMV, biodistribution will be affected especially for multiplication competent GMVs and this can result in infection of cells that are naturally not permissive to the original parental virus. This information is relevant for mapping the dissemination of the recombinant vector. Biodistribution may also influence the time window and routes of shedding of the virus from the recipient and thus, the likelihood of transmission to third parties including vertical transmission and transmission of the vector/recombinant vaccine to other individuals and host species. Further, it is important to map the biodistribution of the virus/vector/recombinant vaccine in immune-compromised individuals given that biodistribution in the immune-compromised is expected to differ from that of the immune-competent individuals. This will provide valuable data on how such recombinant vaccines may affect those with impaired immune system.

#### 3.6.2. Shedding

The environment is exposed to the virus vector/virus-vectored vaccine if it is shed by the vaccine recipient. In this case, hospital personnel, caregivers, non-vacinees and others may be exposed especially if the virus vector is shed via body fluids. When evaluating shedding, the biological properties of the virus vector/virus-vectored vaccine such as biodistribution, the status of the host including immunocompetence, dose and route of administration, sampling frequency and duration of sampling, methods for sample analysis and data interpretation are important considerations. In addition, information on the biological properties of the wild-type strain can provide guidance in shedding evaluations.

Similar to biodistribution, multiplication competence of the virus vector is an important consideration in evaluating shedding given that multiplication competent virus vector may persist in the vaccinee for extended periods of time and can increase in amount leading to higher shedding of infectious particles with an associated likelihood of virus transmission. For such multiplication competent virus vectors, it is important to analyze their molecular variants, which can also influence virus shedding. In general, the basic assumptions in the ERA of multiplication competent virus vectors are that there is a likelihood of spread of the viral vector from the vaccinee into the environment [157]. Immune status of the recipient can affect shedding, a hazard which becomes higher when a multiplication competent virus vector is the case under evaluation. If a virus vector is multiplication incompetent, shedding of the virus is unlikely but evidence should be provided that in such a case, the protein(s) encoded by the inserted transgene(s) has not altered the multiplication incompetence of the vector.

#### 3.6.3. Persistence of Virus Vector in the Environment

The ability of the virus vector to survive after being shed by the vaccinee, as well as the stability of the inserted genetic material in the environment will influence the chance of non-vaccinated organisms being exposed. Persistence of the most commonly used viral vectors in genetically modified (GM) vaccines and gene therapy has been discussed in Baldo et al. [18]. Although poxviruses show high environmental stability, resistance to drying and may remain contagious over a period of several months in ambient temperature [158,159], they are nevertheless sensitive and easily inactivated by all disinfectants commonly approved for surface disinfection [160,161,162].

Persistence of viral particles and DNA in the environment can be affected by several factors (reviewed in [163]), including the presence or absence of proteolytic microorganisms, lipid and protein materials serving as protective shields for the virus particles, pH and temperature (freeze-thaw cycles). Further, presence or absence of DNases, pH and the length of the DNA polymer may affect nucleic acid persistence. There is dearth of information on persistence of virus-vectored vaccine such as recombinant MVA outside the host although persistence in different materials of wild-type viruses commonly employed as vectors is available [163]. Thus, it is often assumed that persistence of GM viruses/vectors is not different from that of the wild-type strains. This ignores the fact that genetic manipulation may have affected some biological properties of the GM virus/vector resulting in a persistence that is different from the corresponding parental virus. An example is where inserted sequences can influence the genome stability of the viral vector, which can result in an improperly packaged, enlarged and unstable genome prone to re-arrangement.

### 3.7. Transmission of Shed Virus Vector and Virus-Vectored Vaccine

In transmission to non-target hosts, persons considered at risk of exposure to virus vector/virus-vectored vaccine are those that come in close contact with the vaccinee. These often are family members, health care workers as well as pets and other animals in close contact with the vaccinee. Knowledge about biodistribution, shedding and the immunological status of the vaccinee close contacts will help in identifying the route of transmission. If the virus-vector is inadvertently transmitted to these individuals, clearance should be effective given that a good proportion of the population already have pre-existing immunity to VACV/MVA. However, should the close contacts have compromised immunity due to old age, very young, sick or are on immunosuppressive drugs, clearance may be inefficient and the consequences of infection might be more significant. In these individuals, interactions (exchange of genetic materials, helper functions of gene products/factors) between GM virus vector and resident natural viruses could generate quasi virus species that are unstable and constantly mutate. This could lead to species with altered epitopes and thus less infectivity [164]. Altered epitope can result in less efficient immune clearance due to weak binding to receptors on the immune cells and can lead to establishment of chronic, latent or persistent infections. A situation in which non-neutralizing antibodies facilitate the infection of other cells (antibody-dependent enhancement of infection) may arise [165,166]. With respect to MVA/MVA-vectored vaccines, the potential for transmission to non-target hosts is low due to its multiplication incompetence in most mammalian cells. However, this observation should be demonstrated *in vitro* in relevant cell cultures, as well as in both immune-competent and immune-deficient animals for each MVA-vectored vaccine. This will confirm that multiplication competence has not been restored in the recombinant MVA vaccine.

### 3.8. Immune Responses: Implications for Biosafety

#### 3.8.1. Anti-Vector Immunity

MVA is considered a safe vaccine against smallpox. However, animal studies showed that higher or multiple doses (boosts) are required to obtain the same anti-VACV antibody response as obtained with a single dose of wild-type VACV [167]. Thus, MVA with higher immunogenicity and recombinant MVA vectors that elicit improved immune response against the heterologous antigen but still reduced virulence are needed. VACV encodes several proteins that are involved in evasion of the host immune response [34,46]. MVA lacks several of these genes and this increases the immunogenicity of the virus [34,46]. VACV infection in mice and humans induces humoral- (antibody secreting B cells or plasma cells) and cell-mediated (CD4+ and CD8+ T cell) responses. Because VACV is used as a vaccine vector, highly attenuated strains such as MVA have been developed. MVA has retained immune stimulatory properties (see [34] for a recent review). Administration of MVA in animal models leads to the induction of type I and type II interferons (IFNs), pro-inflammatory cytokines and chemokines. All are critical mediators of the host anti-viral response. In addition, virus-specific CD4+ and CD8+ T cell responses are developed, including CD8+ memory T cell response [34,168,169]. Despite mutations and major deletions, the MVA genome still encodes several proteins with immunomodulatory functions [46]. Deletion of genes encoding these immunomodulators can increase the immune response to VACV as demonstrated for *A41L* and *B15R* genes. Single removal of these genes resulted in increased viral-specific CD8+ T cell response and enhanced protection against challenging with virulent VACV WR in mice [170,171]. Increased immune responses were also measured after infection with MVA lacking *A35R* or *C12L* [87,88]. These studies suggest that MVA deprived of additional immunomodulatory genes may represent improved vaccine vectors.

The effect of deleting MVA genes in MVA-based vectors expressing foreign antigens on the immune response against these antigens has also been examined. Deletion of single or multiple MVA immunomodulatory genes stimulated the immune response against the foreign proteins expressed by the recombinant MVA mutant [170,172,173]. Alternatively, recombinant MVA vectors expressing host genes encoding immune stimulating proteins may trigger an increased immune response as was shown for MVA expressing granulocyte-macrophage colony-stimulating growth factor (GM-CSF) or C-C Motif Chemokine Ligand 20 (CCL20) in mice [174]. However, in rhesus macaques, high doses of GM-CSF expressed by MVA/GM-CSF inhibited mucosal antibody response and diminished protection elicited by MVA/Simian Immunodeficiency virus (SIV) macaque’s vaccine [175].

Reintroducing VACV genes in MVA to modify the immune response to MVA warrant caution as illustrated by the study of Backes and co-workers [176]. They found that MVA lacking the *C7L* gene (MVA-ΔCL7) could not prevent phosphorylation of eIF2α, an event important in the antiviral response mediated by interferons. Reinsertion of the *K1L* gene, which is involved in NF-κB inhibition and which is absent in MVA, restored eIF2α phosphorylation, indicating the complementary function of the K1L and C7L proteins.

The immunogenicity of MVA cannot only be altered by deletion of immunomodulatory genes, but also by posttranslational modifications of viral structural proteins. Rojas and colleagues found that the glycosylation state of VACV particles had an impact on the immune response [177]. VACV naturally activates Toll-like receptor 2 (TLR2), associated with induction of anti-viral neutralizing antibody response. Deglycosylation of the virus particle blocked TLR2 activation and greatly reduced the production of anti-viral antibodies, but did not affect infectivity. Glycosylated MVA should be used to increase the immune response and cause better protection against poxvirus infection. Vaccination with VACV strains that can multiply in patients with glycosylation defects may result in poorer immune response and poorer protection against poxvirus infection. Strategies used to improve the immunogenicity of (recombinant) MVA besides deletion of immunomodulatory genes include immunization with prime/boost with other viruses or DNA vectors, insertion of genes encoding co-stimulatory molecules (e.g., interleukins, IFNγ), increased expression of the foreign antigen (e.g., promoter strength, codon optimization, terminal signal peptide) and adjuvant (e.g., Glucopyranosyl Lipid A, MF59) (reviewed in [91]. Thus, modulating the expression of MVA genes in these manners can improve the immunogenicity of MVA vaccines and activate immune responses to heterologous antigens. However, the effect of these strategies on the biosafety of MVA including its attenuation, multiplication incompetence, cell and tissue tropism, genome stability, biodistribution and shedding is mostly not investigated.

#### 3.8.2. Effect of Transgenic Protein on Th1-Th2 Response and Cytokine Proliferation

The Th1 cytokine profile is characterized by IFNγ, Interleukin (IL)-2 and IL-12 production, while IL-4, IL-5, IL-6, IL-10 and IL-13 are typical for a Th2 response [178,179]. Infection/immunization with MVA or recombinant MVA can provoke Th1 responses and deletion of MVA genes can further increase these responses. For example, levels of the Th1 cytokines IFN-γ, IL1Β, IL-12 were significantly higher in mice inoculated with MVAΔC12L/A44L-A46R than in animals challenged with MVA [180]. A higher frequency of IFN- γ, TNF-α and IL-2 secreting E3-specific CD8+ T-cells was observed 8 weeks after vaccination with MVA lacking *B15R* compared to MVA [181]. MVA-C, a MVA vector that expressed the envelope protein and the Gag-Pol-Nef from HIV-1 subtype C induced IFN-γ producing CD8+ T cells. The number of these T cells was higher upon infection with a mutant lacking the *F1L* gene [182]. When genes encoding for co-stimulatory molecules and cytokines are inserted into the MVA vector in order to improve immunogenicity, evidence has to be provided to show that the molecules do not predominantly result in a Th2 response. In multiplication competent OPV vectors, insertion of IL-4 resulted in increased pathogenicity of the recombinant virus [183] due to the inhibition of Th1 response [184]. At present, provision of cytokine profile data is not obligatory for the ERA of MVA and MVA-vectored vaccines. However, provision of such a data is important for evaluating the risk of potential reversion of the MVA vector to wild type.

#### 3.8.3. Modulation of Intracellular Signaling Pathways

MVA not only depends on host pathways to successfully generate its progeny, it must also counteract inflammatory, innate and acquired immune response pathways that prevent viral replication [185]. VACV infection resulted in the activation of the Akt signaling pathway [186]. One of the Akt substrates is human cyclic guanosine monophosphate-adenosine monophosphate synthetase (cGAS). Phosphorylation of cGAS inhibits the production of type 1 IFNs and inflammatory cytokines, thereby preventing an antiviral response [187]. Hence, VACV triggered Akt activation helps the virus to evade the host defense mechanism. It is not known whether MVA infection leads to Akt activation. Another pathway that is modulated upon MVA infection is the mitogen-activated protein kinase (MAPK) pathway [188,189,190]. Gedey and colleagues found that MVA activates ERK1/2 in HEK293T cells [191]. This is in contrast with the findings by Schweneker et al. [188] who showed that CVA induced sustained ERK 1/2 activation in HEK293 cells but MVA failed to do so. The authors demonstrated that ERK1/2 activation depended on intact *O1L* gene; a gene fragmented in MVA [46]. The use of different cell lines may explain the discrepancy between the findings of these studies [188,191]. Infection of Hela cells or mouse embryonic fibroblasts (MEFs) with MVA resulted in the upregulation of dual specificity phosphatase 1 (DUSP1), a MAPK dephosphorylating enzyme [192]. Infection of DUSP1 knockout mice with MVA enhanced the production of pro-inflammatory cytokines indicating that DUSP1 is involved in the regulation of innate and adaptive immune responses during poxvirus infection. VACV activates the MAPKs, c-Jun N-terminal protein kinase 1 and 2 (JNK1/2) [189]. Whether MVA can activate the JNK MAPK pathway was not investigated, but inhibition of this pathway causes a significant decline in virus yield in BHK-21 cells [189].

## 4. Future Perspective

### 4.1. Uncertainty and Uncertainty Analysis

Knowledge is always accompanied by uncertainty. Uncertainty with respect to risk characterization refers to (i) lack or incomplete knowledge on the likelihood of an adverse effect occurring and (ii) the lack or insufficient knowledge on the magnitude of the consequences if such an adverse effect occurred. As we have shown in Section 3, there is insufficient knowledge and data on some aspects of MVA biology that are relevant to hazard characterization. Thus, at the very least, there is epistemic uncertainty [193].

Although Directive 2001/18/EC and its supplementary Commission Decision 2002/623/EC recommend identifying uncertainties and analyzing them during ERA of GMOs and medicinal products containing GMOs, the ERA of MVA-vectored vaccines (in peer-reviewed literature and publicly available dossiers submitted to EMA) does not incorporate methodological identification of uncertainty and uncertainty analysis [194]. On the contrary, knowledge about characteristics of the MVA vector that are relevant to ERA is presented as determinate [42], implying that uncertainty is low or negligible [195]. We believe this is a simplification that neither serve the scientific community, nor the research progress in the field. In risk and crisis communication, it is considered a best practice to emphasize and acknowledge uncertainty [196]. Hazard characterization of MVA-vectored vaccines in future should characterize the nature, location and level of the identified uncertainty by employing uncertainty analysis. Uncertainty analysis using different typologies have already been developed for genetically modified (GM) plants, chemical pollutants and nanoparticles [194,197,198] and these could be adapted and applied in the ERA of virus-vectored vaccines including recombinant MVA vaccines.

### 4.2. Worst Case Scenario

Worst-case assumptions that can be tested and eventually rejected or proven are an essential sub-step in the risk characterization of GMOs including genetically modified viruses [199] and plants [200]. The steps used for the ERA of GMVs outlined in Section 1.2 do not have a probabilistic distribution for the frequency of an adverse effect and the level of consequences associated with the adverse effect. Rather, the rating of risk as negligible, low, moderate or high is largely based on weight of evidence (WOE) from peer-reviewed literature and “expert” opinion from scientists commissioned by regulatory agencies like EMA. Much of the published literature on MVA-vectored vaccine addresses vaccine efficacy rather than aspects relevant to ERA. Testing for worst-case scenarios that are falsifiable will reduce uncertainty since a risk analysis where the worst-case scenario is shown not to occur is evidence that the risk of adverse effect is low. In the case of MVA-vectored vaccines, assumptions of worst-case scenarios such as recombination with wild-type OPVs and emergence of more virulent hybrid progenies should be falsified or rejected through experimental investigation in authentic immuno-compromised and immuno-competent animal models.

### 4.3. Conclusions

In this paper, we have highlighted the knowledge gaps and paucity of scientific data on key aspects of MVA biology required for its hazard characterization and presented data obtained in vitro on some worst-case scenarios demonstrating that: (i) MVA productively infects human Caco-2 cells after multiple serial passages in Caco-2 cells and (ii) recombination in vitro between MVA vectored vaccine and a wild type CPXV in a cell line that is semi-permissive to MVA. Provision of empirical data to fill in the gaps in knowledge will improve the hazard characterization of MVA-vectored vaccines resulting in a more robust ERA.

## Figures and Tables

**Table 1:** Susceptibility of mammalian cell lines to modified vaccinia virus Ankara (MVA) strains.

Cell Lines	Species	Multiplication of MVA Strains and Variants ^a^									References
		MVA ^b^	MVA-II/85	MVA-VR1508	MVA-BN	MVA-B	MVA-572	MVA-1721	MVA-574	MVA-LZ	
**MDCK**	Canine; kidney	SP							NP		[45,50]
**Ederm**	Equine; skin								NP		[45]
**RO5R**	Fruit bat Egyptian			P							[58]
**RO5T**	Fruit bat Egyptian			P							[58]
**RO6E**	Fruit bat Egyptian			P							[58]
**CHL**	Hamster Chinese; lung	NP									[50]
**CHO**	Hamster Chinese; ovaries	NP		NP							[50,52]
**BHK-21**	Hamster Syrian; kidney	P		P	P	P				P	[47,49,50,51,52]
**HEK293**	Human, kidney	NP		NP	NP	NP	NP	P		SP	[47,50,51,52,56]
**Hela**	Human; cervix	NP	SP		NP	NP	NP	P	NP	SP	[45,47,50,51,56,57]
**SW839**	Human; kidney	NP									[50]
**TK^−^ 143B**	Human; bone	P	SP		NP		NP	P			[56,57]
**MRC-5**	Human; lung		NP		NP	NP			NP		[45,47,57]
**FS-2**	Human; skin		NP								[57]
**Caco-2**	Human; colorectal			NP							[52]
**FHs74int**	Human; esophagus			NP							[52]
**Hutu-80**	Human; small intestine			NP							[52]
**A549**	Human; lung			SP							[52]
**HaCaT**	Human; skin				NP		SP	P			[56]
**HRT18**	Human; colon								NP		[45]
**Hep-2**	Human; larynx								NP		[45]
**SK 29 MEL 1**	Human; skin									NP	[51]
**LC5**	Human; lung									NP	[51]
**85 HG 66**	Human; brain									NP	[51]
**U138**	Human; brain									NP	[51]
**C8166**	Human; blood (T-cell)									NP	[51]
**HUT 78**	Human; blood (T-cell)									NP	[51]
**SY9287**	Human; blood (B-cell)									NP	[51]
**MA104**	Monkey; kidney								P		[45]
**MIB**	Monkey; blood (B-cell)									NP	[51]
**BSC-1**	Monkey African Green; kidney	SP									[50]
**CV1**	Monkey African Green, kidney	SP, P								P	[50,51]
**Vero**	Monkey African Green; kidney	SP		SP	SP	SP			SP		[45,47,50,52]
**FRhK-4**	Monkey Rhesus; kidney	NP									[51]
**Balb3t3**	Mouse; embryonal fibroblast		NP			NP					[56,57]
**NMULI**	Mouse; glandular epithelial			SP							[52]
**AG101**	Mouse; skin				NP		NP	NP			[56]
**DBT**	Mouse; brain								NP		[45]
**PK(15)**	Pig; kidney	NP		NP							[50,52]
**BEL**	Pig; lung								SP		[45]
**MDBK**	Pig; kidney								NP		[45]
**RK13**	Rabbit; kidney	NP		NP					NP		[45,50,52]
**RAB-9**	Rabbit; skin	NP									[50]
**SIRC**	Rabbit; cornea	NP									[50]
**IEC-6**	Rat; small intestine			P		SP					[47,52]
**H4IIE**	Rat; liver			NP							[52]

^a^ Virus multiplication is the ratio of output to input virus titer as defined by Carroll et al. [50]; ^b^ All MVA whose strain, variant or passage number was not stated in the relevant publication. P: permissive, SP: semi-permissive, NP: non-permissive.

## References

[1] Ura T., Okuda K., Shimada M. (2014). Developments in viral vector-based vaccines. Vaccines.

[2] Ramezanpour B., Haan I., Osterhaus A., Claassen E. (2016). Vector-based genetically modified vaccines: Exploiting Jenner’s legacy. Vaccine.

[3] Gilbert S.C. (2013). Clinical development of Modified Vaccinia virus Ankara vaccines. Vaccine.

[4] Cooney E.L., Collier A.C., Greenberg P.D., Coombs R.W., Zarling J., Arditti D.E., Hoffman M.C., Hu S.L., Corey L. (1991). Safety of and immunological response to a recombinant vaccinia virus vaccine expressing HIV envelope glycoprotein. Lancet.

[5] Arnberg N. (2009). Adenovirus receptors: Implications for tropism, treatment and targeting. Rev. Med. Virol..

[6] Roberts D.M., Nanda A., Havenga M.J., Abbink P., Lynch D.M., Ewald B.A., Liu J., Thorner A.R., Swanson P.E., Gorgone D.A. (2006). Hexon-chimaeric adenovirus serotype 5 vectors circumvent pre-existing anti-vector immunity. Nature.

[7] Abe S., Okuda K., Ura T., Kondo A., Yoshida A., Yoshizaki S., Mizuguchi H., Klinman D., Shimada M. (2009). Adenovirus type 5 with modified hexons induces robust transgene-specific immune responses in mice with pre-existing immunity against adenovirus type 5. J. Gene Med..

[8] Ura T., Yoshida A., Xin K.Q., Yoshizaki S., Yashima S., Abe S., Mizuguchi H., Okuda K. (2009). Designed recombinant adenovirus type 5 vector induced envelope-specific CD8(+) cytotoxic T lymphocytes and cross-reactive neutralizing antibodies against human immunodeficiency virus type 1. J. Gene Med..

[9] Donsante A., Vogler C., Muzyczka N., Crawford J.M., Barker J., Flotte T., Campbell-Thompson M., Daly T., Sands M.S. (2001). Observed incidence of tumorigenesis in long-term rodent studies of rAAV vectors. Gene Ther..

[10] Lentz T.B., Gray S.J., Samulski R.J. (2012). Viral vectors for gene delivery to the central nervous system. Neurobiol. Dis..

[11] Li Z., Dullmann J., Schiedlmeier B., Schmidt M., von Kalle C., Meyer J., Forster M., Stocking C., Wahlers A., Frank O. (2002). Murine leukemia induced by retroviral gene marking. Science.

[12] Qasim W., Gaspar H.B., Thrasher A.J. (2004). Gene therapy for severe combined immune deficiency. Expert Rev. Mol. Med..

[13] Moriya C., Horiba S., Kurihara K., Kamada T., Takahara Y., Inoue M., Iida A., Hara H., Shu T., Hasegawa M. (2011). Intranasal Sendai viral vector vaccination is more immunogenic than intramuscular under pre-existing anti-vector antibodies. Vaccine.

[14] (2001). Directive 2001/18/EC of the European Parliament and of the Council of 12 March on the deliberate release into the environment of genetically modified organisms and repealing Council Directive 90/220/EEC. European Parliament and Council.

[15] Anson D.S. (2004). The use of retroviral vectors for gene therapy—What are the risks? A review of retroviral pathogenesis and its relevance to retroviral vector-mediated gene delivery. Genet. Vaccines Ther..

[16] Auman J.T. (2010). Gene therapy: Have the risks associated with viral vectors been solved?. Curr. Opin. Mol. Ther..

[17] Verheust C., Goossens M., Pauwels K., Breyer D. (2012). Biosafety aspects of modified vaccinia virus Ankara (MVA)-based vectors used for gene therapy or vaccination. Vaccine.

[18] Baldo A., van den Akker E., Bergmans H.E., Lim F., Pauwels K. (2013). General considerations on the biosafety of virus-derived vectors used in gene therapy and vaccination. Curr. Gene Ther..

[19] Goossens M., Pauwels K., Willemarck N., Breyer D. (2013). Environmental risk assessment of clinical trials involving modified vaccinia virus Ankara (MVA)-based vectors. Curr. Gene Ther..

[20] Moss B., Roizman B., Howley P., Straus S., Martin M., DE G., Lamb R., Knipe D. (2001). Poxviridae: The Viruses and Their Replication. Fields Virology.

[21] Fenner F.H., Hender D.A., Arita I., Jesek J., Ladnyi I.D. (1988). Smallpox and Its Eradication.

[22] Parrino J., Graham B.S. (2006). Smallpox vaccines: Past, present and future. J. Allergy Clin. Immunol..

[23] Geddes A.M. (2006). The history of smallpox. Clin. Dermatol..

[24] Drewitt F.D. (1933). The Life of Edward Jenner.

[25] Jenner E. (2001). The Three Original Publications on Vaccination Agianst Smallpox.

[26] Sanchez-Sampedro L., Perdiguero B., Mejias-Perez E., Garcia-Arriaza J., Di Pilato M., Esteban M. (2015). The evolution of poxvirus vaccines. Viruses.

[27] Koplow D.A. (2003). Smallpox: The Fight to Eradicate a Global Scourge.

[28] Panicali D., Paoletti E. (1982). Construction of poxviruses as cloning vectors: Insertion of the thymidine kinase gene from herpes simplex virus into the DNA of infectious vaccinia virus. Proc. Natl. Acad. Sci. USA.

[29] Mackett M., Smith G.L., Moss B. (1982). Vaccinia virus: A selectable eukaryotic cloning and expression vector. Proc. Natl. Acad. Sci. USA.

[30] Whitley R.J. (2003). Smallpox: A potential agent of bioterrorism. Antivir. Res..

[31] Damaso C.R., Esposito J.J., Condit R.C., Moussatche N. (2000). An emergent poxvirus from humans and cattle in Rio de Janeiro State: Cantagalo virus may derive from Brazilian smallpox vaccine. Virology.

[32] Vorou R.M., Papavassiliou V.G., Pierroutsakos I.N. (2008). Cowpox virus infection: An emerging health threat. Curr. Opin. Infect. Dis..

[33] Reed K.D., Melski J.W., Graham M.B., Regnery R.L., Sotir M.J., Wegner M.V., Kazmierczak J.J., Stratman E.J., Li Y., Fairley J.A. (2004). The detection of monkeypox in humans in the Western Hemisphere. N. Engl. J. Med..

[34] Volz A., Sutter G. (2017). Modified vaccinia virus ankara: History, value in basic research and current perspectives for vaccine development. Adv. Virus Res..

[35] Liu Z., Ravindranathan R., Kalinski P., Guo Z.S., Bartlett D.L. (2017). Rational combination of oncolytic vaccinia virus and PD-L1 blockade works synergistically to enhance therapeutic efficacy. Nat. Commun..

[36] Izzi V., Buler M., Masuelli L., Giganti M.G., Modesti A., Bei R. (2014). Poxvirus-based vaccines for cancer immunotherapy: New insights from combined cytokines/co-stimulatory molecules delivery and “uncommon” strains. Anticancer Agents Med. Chem..

[37] Rupprecht C.E., Wiktor T.J., Johnston D.H., Hamir A.N., Dietzschold B., Wunner W.H., Glickman L.T., Koprowski H. (1986). Oral immunization and protection of raccoons (Procyon lotor) with a vaccinia-rabies glycoprotein recombinant virus vaccine. Proc. Natl. Acad. Sci. USA.

[38] Muller T.F., Schroder R., Wysocki P., Mettenleiter T.C., Freuling C.M. (2015). Spatio-temporal use of oral rabies vaccines in fox rabies elimination programmes in Europe. PLoS Negl. Trop. Dis..

[39] Casey C.G., Iskander J.K., Roper M.H., Mast E.E., Wen X.J., Torok T.J., Chapman L.E., Swerdlow D.L., Morgan J., Heffelfinger J.D. (2005). Adverse events associated with smallpox vaccination in the United States, January–October 2003. JAMA.

[40] Mempel M., Isa G., Klugbauer N., Meyer H., Wildi G., Ring J., Hofmann F., Hofmann H. (2003). Laboratory acquired infection with recombinant vaccinia virus containing an immunomodulating construct. J. Investig. Dermatol..

[41] Draper S.J., Heeney J.L. (2010). Viruses as vaccine vectors for infectious diseases and cancer. Nat. Rev. Microbiol..

[42] Cottingham M.G., Carroll M.W. (2013). Recombinant MVA vaccines: Dispelling the myths. Vaccine.

[43] Altenburg A.F., Kreijtz J.H., de Vries R.D., Song F., Fux R., Rimmelzwaan G.F., Sutter G., Volz A. (2014). Modified vaccinia virus ankara (MVA) as production platform for vaccines against influenza and other viral respiratory diseases. Viruses.

[44] Mayr A., Stickl H., Muller H.K., Danner K., Singer H. (1978). The smallpox vaccination strain MVA: Marker, genetic structure, experience gained with the parenteral vaccination and behavior in organisms with a debilitated defence mechanism (author’s transl). Zent. Bakteriol. B.

[45] Meyer H., Sutter G., Mayr A. (1991). Mapping of deletions in the genome of the highly attenuated vaccinia virus MVA and their influence on virulence. J. Gen. Virol..

[46] Antoine G., Scheiflinger F., Dorner F., Falkner F.G. (1998). The complete genomic sequence of the modified vaccinia Ankara strain: Comparison with other orthopoxviruses. Virology.

[47] Meisinger-Henschel C., Spath M., Lukassen S., Wolferstatter M., Kachelriess H., Baur K., Dirmeier U., Wagner M., Chaplin P., Suter M. (2010). Introduction of the six major genomic deletions of modified vaccinia virus Ankara (MVA) into the parental vaccinia virus is not sufficient to reproduce an MVA-like phenotype in cell culture and in mice. J. Virol..

[48] Stickl H., Hochstein-Mintzel V., Mayr A., Huber H.C., Schafer H., Holzner A. (1974). MVA vaccination against smallpox: Clinical tests with an attenuated live vaccinia virus strain (MVA) (author’s transl). Dtsch. Med. Wochenschr..

[49] Sutter G., Moss B. (1992). Nonreplicating vaccinia vector efficiently expresses recombinant genes. Proc. Natl. Acad. Sci. USA.

[50] Carroll M.W., Moss B. (1997). Host range and cytopathogenicity of the highly attenuated MVA strain of vaccinia virus: Propagation and generation of recombinant viruses in a nonhuman mammalian cell line. Virology.

[51] Drexler I., Heller K., Wahren B., Erfle V., Sutter G. (1998). Highly attenuated modified vaccinia virus Ankara replicates in baby hamster kidney cells, a potential host for virus propagation, but not in various human transformed and primary cells. J. Gen. Virol..

[52] Okeke M.I., Nilssen O., Traavik T. (2006). Modified vaccinia virus Ankara multiplies in rat IEC-6 cells and limited production of mature virions occurs in other mammalian cell lines. J. Gen. Virol..

[53] Meyer H. (2013). Summary Report on First, Second and Third Generation Smallpox Vaccines.

[54] Slifka M.K. (2005). The Future of smallpox vaccination: Is MVA the key?. Med. Immunol..

[55] Earl P.L., Americo J.L., Wyatt L.S., Eller L.A., Whitbeck J.C., Cohen G.H., Eisenberg R.J., Hartmann C.J., Jackson D.L., Kulesh D.A. (2004). Immunogenicity of a highly attenuated MVA smallpox vaccine and protection against monkeypox. Nature.

[56] Suter M., Meisinger-Henschel C., Tzatzaris M., Hulsemann V., Lukassen S., Wulff N.H., Hausmann J., Howley P., Chaplin P. (2009). Modified vaccinia Ankara strains with identical coding sequences actually represent complex mixtures of viruses that determine the biological properties of each strain. Vaccine.

[57] Blanchard T.J., Alcami A., Andrea P., Smith G.L. (1998). Modified vaccinia virus Ankara undergoes limited replication in human cells and lacks several immunomodulatory proteins: Implications for use as a human vaccine. J. Gen. Virol..

[58] Jordan I., Horn D., Oehmke S., Leendertz F.H., Sandig V. (2009). Cell lines from the Egyptian fruit bat are permissive for modified vaccinia Ankara. Virus Res..

[59] Dorrell L., Williams P., Suttill A., Brown D., Roberts J., Conlon C., Hanke T., McMichael A. (2007). Safety and tolerability of recombinant modified vaccinia virus Ankara expressing an HIV-1 gag/multiepitope immunogen (MVA.HIVA) in HIV-1-infected persons receiving combination antiretroviral therapy. Vaccine.

[60] Hanke T., McMichael A.J., Dennis M.J., Sharpe S.A., Powell L.A., McLoughlin L., Crome S.J. (2005). Biodistribution and persistence of an MVA-vectored candidate HIV vaccine in SIV-infected rhesus macaques and SCID mice. Vaccine.

[61] Darsow U., Sbornik M., Rombold S., Katzer K., von Sonnenburg F., Behrendt H., Ring J. (2016). Long-term safety of replication-defective smallpox vaccine (MVA-BN) in atopic eczema and allergic rhinitis. J. Eur. Acad. Dermatol. Venereol..

[62] Overton E.T., Stapleton J., Frank I., Hassler S., Goepfert P.A., Barker D., Wagner E., von Krempelhuber A., Virgin G., Meyer T.P. (2015). Safety and Immunogenicity of Modified Vaccinia Ankara-Bavarian Nordic Smallpox Vaccine in Vaccinia-Naive and Experienced Human Immunodeficiency Virus-Infected Individuals: An Open-Label, Controlled Clinical Phase II Trial. Open Forum Infect. Dis..

[63] Earl P.L., Americo J.L., Wyatt L.S., Espenshade O., Bassler J., Gong K., Lin S., Peters E., Rhodes L., Spano Y.E. (2008). Rapid protection in a monkeypox model by a single injection of a replication-deficient vaccinia virus. Proc. Natl. Acad. Sci USA.

[64] Guerra S., Gonzalez J.M., Climent N., Reyburn H., Lopez-Fernandez L.A., Najera J.L., Gomez C.E., Garcia F., Gatell J.M., Gallart T. (2010). Selective induction of host genes by MVA-B, a candidate vaccine against HIV/AIDS. J. Virol..

[65] Joachim A., Nilsson C., Aboud S., Bakari M., Lyamuya E.F., Robb M.L., Marovich M.A., Earl P., Moss B., Ochsenbauer C. (2015). Potent functional antibody responses elicited by HIV-I DNA priming and boosting with heterologous HIV-1 recombinant MVA in healthy Tanzanian adults. PLoS ONE.

[66] Munseri P.J., Kroidl A., Nilsson C., Joachim A., Geldmacher C., Mann P., Moshiro C., Aboud S., Lyamuya E., Maboko L. (2015). Priming with a simplified intradermal HIV-1 DNA vaccine regimen followed by boosting with recombinant HIV-1 MVA vaccine is safe and immunogenic: A phase IIa randomized clinical trial. PLoS ONE.

[67] Nilsson C., Godoy-Ramirez K., Hejdeman B., Brave A., Gudmundsdotter L., Hallengard D., Currier J.R., Wieczorek L., Hasselrot K., Earl P.L. (2014). Broad and potent cellular and humoral immune responses after a second late HIV-modified vaccinia virus ankara vaccination in HIV-DNA-primed and HIV-modified vaccinia virus Ankara-boosted Swedish vaccinees. AIDS Res. Hum. Retrovir..

[68] Tameris M.D., Hatherill M., Landry B.S., Scriba T.J., Snowden M.A., Lockhart S., Shea J.E., McClain J.B., Hussey G.D., Hanekom W.A. (2013). Safety and efficacy of MVA85A, a new tuberculosis vaccine, in infants previously vaccinated with BCG: A randomised, placebo-controlled phase 2b trial. Lancet.

[69] Hodgson S.H., Ewer K.J., Bliss C.M., Edwards N.J., Rampling T., Anagnostou N.A., de Barra E., Havelock T., Bowyer G., Poulton I.D. (2015). Evaluation of the efficacy of ChAd63-MVA vectored vaccines expressing circumsporozoite protein and ME-TRAP against controlled human malaria infection in malaria-naive individuals. J. Infect. Dis..

[70] Biswas S., Choudhary P., Elias S.C., Miura K., Milne K.H., de Cassan S.C., Collins K.A., Halstead F.D., Bliss C.M., Ewer K.J. (2014). Assessment of humoral immune responses to blood-stage malaria antigens following ChAd63-MVA immunization, controlled human malaria infection and natural exposure. PLoS ONE.

[71] Sebastian S., Gilbert S.C. (2016). Recombinant modified vaccinia virus Ankara-based malaria vaccines. Expert Rev. Vaccin..

[72] Milligan I.D., Gibani M.M., Sewell R., Clutterbuck E.A., Campbell D., Plested E., Nuthall E., Voysey M., Silva-Reyes L., McElrath M.J. (2016). Safety and Immunogenicity of Novel Adenovirus Type 26- and Modified Vaccinia Ankara-Vectored Ebola Vaccines: A Randomized Clinical Trial. JAMA.

[73] Tapia M.D., Sow S.O., Lyke K.E., Haidara F.C., Diallo F., Doumbia M., Traore A., Coulibaly F., Kodio M., Onwuchekwa U. (2016). Use of ChAd3-EBO-Z Ebola virus vaccine in Malian and US adults and boosting of Malian adults with MVA-BN-Filo: A phase 1, single-blind, randomised trial, a phase 1b, open-label and double-blind, dose-escalation trial and a nested, randomised, double-blind, placebo-controlled trial. Lancet Infect. Dis..

[74] Kreijtz J.H., Goeijenbier M., Moesker F.M., van den Dries L., Goeijenbier S., De Gruyter H.L., Lehmann M.H., Mutsert G., van de Vijver D.A., Volz A. (2014). Safety and immunogenicity of a modified-vaccinia-virus-Ankara-based influenza A H5N1 vaccine: A randomised, double-blind phase 1/2a clinical trial. Lancet Infect. Dis..

[75] Lillie P.J., Berthoud T.K., Powell T.J., Lambe T., Mullarkey C., Spencer A.J., Hamill M., Peng Y., Blais M.E., Duncan C.J. (2012). Preliminary assessment of the efficacy of a T-cell-based influenza vaccine, MVA-NP+M1, in humans. Clin. Infect. Dis..

[76] Cavenaugh J.S., Awi D., Mendy M., Hill A.V., Whittle H., McConkey S.J. (2011). Partially randomized, non-blinded trial of DNA and MVA therapeutic vaccines based on hepatitis B virus surface protein for chronic HBV infection. PLoS ONE.

[77] Volz A., Sutter G. (2013). Protective efficacy of Modified Vaccinia virus Ankara in preclinical studies. Vaccine.

[78] Cappuccini F., Pollock E., Stribbling S., Hill A.V.S., Redchenko I. (2017). 5T4 oncofoetal glycoprotein: An old target for a novel prostate cancer immunotherapy. Oncotarget.

[79] Husseini F., Delord J.P., Fournel-Federico C., Guitton J., Erbs P., Homerin M., Halluard C., Jemming C., Orange C., Limacher J.M. (2017). Vectorized gene therapy of liver tumors: Proof-of-concept of TG4023 (MVA-FCU1) in combination with flucytosine. Ann. Oncol..

[80] Schaedler E., Remy-Ziller C., Hortelano J., Kehrer N., Claudepierre M.C., Gatard T., Jakobs C., Preville X., Carpentier A.F., Rittner K. (2017). Sequential administration of a MVA-based MUC1 cancer vaccine and the TLR9 ligand Litenimod (Li28) improves local immune defense against tumors. Vaccine.

[81] Rowe J., Cen P. (2014). TroVax in colorectal cancer. Hum. Vaccin. Immunother..

[82] Alberca B., Bachanek-Bankowska K., Cabana M., Calvo-Pinilla E., Viaplana E., Frost L., Gubbins S., Urniza A., Mertens P., Castillo-Olivares J. (2014). Vaccination of horses with a recombinant modified vaccinia Ankara virus (MVA) expressing African horse sickness (AHS) virus major capsid protein VP2 provides complete clinical protection against challenge. Vaccine.

[83] Haagmans B.L., van den Brand J.M., Raj V.S., Volz A., Wohlsein P., Smits S.L., Schipper D., Bestebroer T.M., Okba N., Fux R. (2016). An orthopoxvirus-based vaccine reduces virus excretion after MERS-CoV infection in dromedary camels. Science.

[84] Lopera-Madrid J., Osorio J.E., He Y., Xiang Z., Adams L.G., Laughlin R.C., Mwangi W., Subramanya S., Neilan J., Brake D. (2017). Safety and immunogenicity of mammalian cell derived and Modified Vaccinia Ankara vectored African swine fever subunit antigens in swine. Vet. Immunol. Immunopathol..

[85] Del Medico Zajac M.P., Zanetti F.A., Esusy M.S., Federico C.R., Zabal O., Valera A.R., Calamante G. (2017). Induction of Both Local Immune Response in Mice and Protection in a Rabbit Model by Intranasal Immunization with Modified Vaccinia Ankara Virus Expressing a Secreted Form of Bovine Herpesvirus 1 Glycoprotein D. Viral. Immunol..

[86] Volz A., Fux R., Langenmayer M.C., Sutter G. (2015). Modified vaccinia virus ankara (MVA)—Development as recombinant vaccine and prospects for use in veterinary medicine. Berl. Munch. Tierarztl. Wochenschr..

[87] Falivene J., del Medico Zajac M.P., Pascutti M.F., Rodriguez A.M., Maeto C., Perdiguero B., Gomez C.E., Esteban M., Calamante G., Gherardi M.M. (2012). Improving the MVA vaccine potential by deleting the viral gene coding for the IL-18 binding protein. PLoS ONE.

[88] Rehm K.E., Roper R.L. (2011). Deletion of the A35 gene from Modified Vaccinia Virus Ankara increases immunogenicity and isotype switching. Vaccine.

[89] Legrand F.A., Verardi P.H., Jones L.A., Chan K.S., Peng Y., Yilma T.D. (2004). Induction of potent humoral and cell-mediated immune responses by attenuated vaccinia virus vectors with deleted serpin genes. J. Virol..

[90] Mandl S.J., Rountree R.B., Dalpozzo K., Do L., Lombardo J.R., Schoonmaker P.L., Dirmeier U., Steigerwald R., Giffon T., Laus R. (2012). Immunotherapy with MVA-BN(R)-HER2 induces HER-2-specific Th1 immunity and alters the intratumoral balance of effector and regulatory T cells. Cancer Immunol. Immunother..

[91] Garcia-Arriaza J., Esteban M. (2014). Enhancing poxvirus vectors vaccine immunogenicity. Hum. Vaccin. Immunother..

[92] Baur K., Brinkmann K., Schweneker M., Patzold J., Meisinger-Henschel C., Hermann J., Steigerwald R., Chaplin P., Suter M., Hausmann J. (2010). Immediate-early expression of a recombinant antigen by modified vaccinia virus ankara breaks the immunodominance of strong vector-specific B8R antigen in acute and memory CD8 T-cell responses. J. Virol..

[93] Wennier S.T., Brinkmann K., Steinhausser C., Maylander N., Mnich C., Wielert U., Dirmeier U., Hausmann J., Chaplin P., Steigerwald R. (2013). A novel naturally occurring tandem promoter in modified vaccinia virus ankara drives very early gene expression and potent immune responses. PLoS ONE.

[94] Di Pilato M., Mejias-Perez E., Gomez C.E., Perdiguero B., Sorzano C.O., Esteban M. (2013). New vaccinia virus promoter as a potential candidate for future vaccines. J. Gen. Virol..

[95] Hansen H., Okeke M.I., Nilssen O., Traavik T. (2004). Recombinant viruses obtained from co-infection in vitro with a live vaccinia-vectored influenza vaccine and a naturally occurring cowpox virus display different plaque phenotypes and loss of the transgene. Vaccine.

[96] Okeke M.I., Nilssen O., Moens U., Tryland M., Traavik T. (2009). In vitro host range, multiplication and virion forms of recombinant viruses obtained from co-infection in vitro with a vaccinia-vectored influenza vaccine and a naturally occurring cowpox virus isolate. Virol. J..

[97] European Medicines Agency. http://www.ema.europa.eu/ema/.

[98] Sutter G., Ramsey-Ewing A., Rosales R., Moss B. (1994). Stable expression of the vaccinia virus K1L gene in rabbit cells complements the host range defect of a vaccinia virus mutant. J. Virol..

[99] Wyatt L.S., Carroll M.W., Czerny C.P., Merchlinsky M., Sisler J.R., Moss B. (1998). Marker rescue of the host range restriction defects of modified vaccinia virus Ankara. Virology.

[100] Mayr A., Munz E. (1964). Changes in the vaccinia virus through continuing passages in chick embryo fibroblast cultures. Zent. Bakteriol. Orig..

[101] Qin L., Upton C., Hazes B., Evans D.H. (2011). Genomic analysis of the vaccinia virus strain variants found in Dryvax vaccine. J. Virol..

[102] Burgers W.A., Shephard E., Monroe J.E., Greenhalgh T., Binder A., Hurter E., van Harmelen J.H., Williamson C., Williamson A.L. (2008). Construction, characterization and immunogenicity of a multigene modified vaccinia Ankara (MVA) vaccine based on HIV type 1 subtype C. AIDS Res. Hum. Retrovir..

[103] Wyatt L.S., Belyakov I.M., Earl P.L., Berzofsky J.A., Moss B. (2008). Enhanced cell surface expression, immunogenicity and genetic stability resulting from a spontaneous truncation of HIV Env expressed by a recombinant MVA. Virology.

[104] Wang Z., Martinez J., Zhou W., La Rosa C., Srivastava T., Dasgupta A., Rawal R., Li Z., Britt W.J., Diamond D. (2010). Modified H5 promoter improves stability of insert genes while maintaining immunogenicity during extended passage of genetically engineered MVA vaccines. Vaccine.

[105] Timm A., Enzinger C., Felder E., Chaplin P. (2006). Genetic stability of recombinant MVA-BN. Vaccine.

[106] Manuel E.R., Wang Z., Li Z., La Rosa C., Zhou W., Diamond D.J. (2010). Intergenic region 3 of modified vaccinia ankara is a functional site for insert gene expression and allows for potent antigen-specific immune responses. Virology.

[107] Alharbi N.K., Spencer A.J., Salman A.M., Tully C.M., Chinnakannan S.K., Lambe T., Yamaguchi Y., Morris S.J., Orubu T., Draper S.J. (2016). Enhancing cellular immunogenicity of MVA-vectored vaccines by utilizing the F11L endogenous promoter. Vaccine.

[108] Orubu T., Alharbi N.K., Lambe T., Gilbert S.C., Cottingham M.G. (2012). Expression and cellular immunogenicity of a transgenic antigen driven by endogenous poxviral early promoters at their authentic loci in MVA. PLoS ONE.

[109] Di Pilato M., Sanchez-Sampedro L., Mejias-Perez E., Sorzano C.O., Esteban M. (2015). Modification of promoter spacer length in vaccinia virus as a strategy to control the antigen expression. J. Gen. Virol..

[110] Chakrabarti S., Sisler J.R., Moss B. (1997). Compact, synthetic, vaccinia virus early/late promoter for protein expression. Biotechniques.

[111] Wyatt L.S., Earl P.L., Xiao W., Americo J.L., Cotter C.A., Vogt J., Moss B. (2009). Elucidating and minimizing the loss by recombinant vaccinia virus of human immunodeficiency virus gene expression resulting from spontaneous mutations and positive selection. J. Virol..

[112] Franke A., Pfaff F., Jenckel M., Hoffmann B., Hoper D., Antwerpen M., Meyer H., Beer M., Hoffmann D. (2017). Classification of Cowpox Viruses into Several Distinct Clades and Identification of a Novel Lineage. Viruses.

[113] Meyer H., Schay C., Mahnel H., Pfeffer M. (1999). Characterization of orthopoxviruses isolated from man and animals in Germany. Arch. Virol..

[114] Dabrowski P.W., Radonic A., Kurth A., Nitsche A. (2013). Genome-wide comparison of cowpox viruses reveals a new clade related to Variola virus. PLoS ONE.

[115] Mauldin M.R., Antwerpen M., Emerson G.L., Li Y., Zoeller G., Carroll D.S., Meyer H. (2017). Cowpox virus: What’s in a Name?. Viruses.

[116] Kaysser P., von Bomhard W., Dobrzykowski L., Meyer H. (2010). Genetic diversity of feline cowpox virus, Germany 2000–2008. Vet. Microbiol..

[117] Chantrey J., Meyer H., Baxby D., Begon M., Bown K.J., Hazel S.M., Jones T., Montgomery W.I., Bennett M. (1999). Cowpox: Reservoir hosts and geographic range. Epidemiol. Infect..

[118] Crouch A.C., Baxby D., McCracken C.M., Gaskell R.M., Bennett M. (1995). Serological evidence for the reservoir hosts of cowpox virus in British wildlife. Epidemiol. Infect..

[119] Hansen H., Okeke M.I., Nilssen O., Traavik T. (2009). Comparison and phylogenetic analysis of cowpox viruses isolated from cats and humans in Fennoscandia. Arch. Virol..

[120] Tryland M., Sandvik T., Arnemo J.M., Stuve G., Olsvik O., Traavik T. (1998). Antibodies against orthopoxviruses in wild carnivores from Fennoscandia. J. Wildl. Dis..

[121] Tryland M., Sandvik T., Mehl R., Bennett M., Traavik T., Olsvik O. (1998). Serosurvey for orthopoxviruses in rodents and shrews from Norway. J. Wildl. Dis..

[122] Tryland M., Okeke M.I., Af Segerstad C.H., Morner T., Traavik T., Ryser-Degiorgis M.P. (2011). Orthopoxvirus DNA in Eurasian lynx, Sweden. Emerg. Infect. Dis..

[123] Okeke M.I., Hansen H., Traavik T. (2012). A naturally occurring cowpox virus with an ectromelia virus A-type inclusion protein gene displays atypical A-type inclusions. Infect. Genet. Evol..

[124] Kinnunen P.M., Henttonen H., Hoffmann B., Kallio E.R., Korthase C., Laakkonen J., Niemimaa J., Palva A., Schlegel M., Ali H.S. (2011). Orthopox virus infections in Eurasian wild rodents. Vector Borne Zoonotic Dis..

[125] Emerson G.L., Li Y., Frace M.A., Olsen-Rasmussen M.A., Khristova M.L., Govil D., Sammons S.A., Regnery R.L., Karem K.L., Damon I.K. (2009). The phylogenetics and ecology of the orthopoxviruses endemic to North America. PLoS ONE.

[126] Springer Y.P., Hsu C.H., Werle Z.R., Olson L.E., Cooper M.P., Castrodale L.J., Fowler N., McCollum A.M., Goldsmith C.S., Emerson G.L. (2017). Novel Orthopoxvirus Infection in an Alaska Resident. Clin. Infect. Dis..

[127] Fleischauer C., Upton C., Victoria J., Jones G.J., Roper R.L. (2015). Genome sequence and comparative virulence of raccoonpox virus: The first North American poxvirus sequence. J. Gen. Virol..

[128] Smithson C., Tang N., Sammons S., Frace M., Batra D., Li Y., Emerson G.L., Carroll D.S., Upton C. (2017). The genomes of three North American orthopoxviruses. Virus Genes.

[129] Miranda J.B., Borges I.A., Campos S.P.S., Vieira F.N., de Azara T.M.F., Marques F.A., Costa G.B., Luis A., de Oliveira J.S., Ferreira P.C.P. (2017). Serologic and Molecular Evidence of Vaccinia Virus Circulation among Small Mammals from Different Biomes, Brazil. Emerg. Infect. Dis..

[130] Costa G.B., Augusto L.T., Leite J.A., Ferreira P.C., Bonjardim C.A., Abrahao J.S., Kroon E.G., Moreno E.C., Trindade Gde S. (2016). Seroprevalence of Orthopoxvirus in rural Brazil: Insights into anti-OPV immunity status and its implications for emergent zoonotic OPV. Virol. J..

[131] Oliveira G., Assis F., Almeida G., Albarnaz J., Lima M., Andrade A.C., Calixto R., Oliveira C., Diomedes Neto J., Trindade G. (2015). From lesions to viral clones: Biological and molecular diversity amongst autochthonous Brazilian vaccinia virus. Viruses.

[132] Moussatche N., Damaso C.R., McFadden G. (2008). When good vaccines go wild: Feral Orthopoxvirus in developing countries and beyond. J. Infect. Dev. Ctries.

[133] Singh R.K., Hosamani M., Balamurugan V., Bhanuprakash V., Rasool T.J., Yadav M.P. (2007). Buffalopox: An emerging and re-emerging zoonosis. Anim. Health Res. Rev..

[134] Bhanuprakash V., Venkatesan G., Balamurugan V., Hosamani M., Yogisharadhya R., Gandhale P., Reddy K.V., Damle A.S., Kher H.N., Chandel B.S. (2010). Zoonotic infections of buffalopox in India. Zoonoses Public Health.

[135] Bera B.C., Shanmugasundaram K., Barua S., Venkatesan G., Virmani N., Riyesh T., Gulati B.R., Bhanuprakash V., Vaid R.K., Kakker N.K. (2011). Zoonotic cases of camelpox infection in India. Vet. Microbiol..

[136] Afolabi M.O., Tiono A.B., Adetifa U.J., Yaro J.B., Drammeh A., Nebie I., Bliss C., Hodgson S.H., Anagnostou N.A., Sanou G.S. (2016). Safety and Immunogenicity of ChAd63 and MVA ME-TRAP in West African Children and Infants. Mol. Ther..

[137] Mensah V.A., Gueye A., Ndiaye M., Edwards N.J., Wright D., Anagnostou N.A., Syll M., Ndaw A., Abiola A., Bliss C. (2016). Safety, Immunogenicity and Efficacy of Prime-Boost Vaccination with ChAd63 and MVA Encoding ME-TRAP against Plasmodium falciparum Infection in Adults in Senegal. PLoS ONE.

[138] Excler J.L., Michael N.L. (2016). Lessons from HIV-1 vaccine efficacy trials. Curr. Opin. HIV AIDS.

[139] Brochier B.M., Languet B., Blancou J., Kieny M.P., Lecocq J.P., Costy F., Desmettre P., Pastoret P.P. (1988). Use of recombinant vaccinia-rabies virus for oral vaccination of fox cubs (*Vulpes vulpes*, L) against rabies. Vet. Microbiol..

[140] Freuling C.M., Hampson K., Selhorst T., Schroder R., Meslin F.X., Mettenleiter T.C., Muller T. (2013). The elimination of fox rabies from Europe: Determinants of success and lessons for the future. Philos. Trans. R. Soc. Lond. B Biol. Sci..

[141] Ball L.A. (1987). High-frequency homologous recombination in vaccinia virus DNA. J. Virol..

[142] Fathi Z., Dyster L.M., Seto J., Condit R.C., Niles E.G. (1991). Intragenic and intergenic recombination between temperature-sensitive mutants of vaccinia virus. J. Gen. Virol..

[143] Block W., Upton C., McFadden G. (1985). Tumorigenic poxviruses: Genomic organization of malignant rabbit virus, a recombinant between Shope fibroma virus and myxoma virus. Virology.

[144] Qin L., Evans D.H. (2014). Genome scale patterns of recombination between coinfecting vaccinia viruses. J. Virol..

[145] Staib C., Drexler I., Sutter G. (2004). Construction and isolation of recombinant MVA. Methods Mol. Biol..

[146] Lin Y.C., Evans D.H. (2010). Vaccinia virus particles mix inefficiently and in a way that would restrict viral recombination, in co-infected cells. J. Virol..

[147] Doceul V., Hollinshead M., van der Linden L., Smith G.L. (2010). Repulsion of superinfecting virions: A mechanism for rapid virus spread. Science.

[148] Laliberte J.P., Moss B. (2014). A novel mode of poxvirus superinfection exclusion that prevents fusion of the lipid bilayers of viral and cellular membranes. J. Virol..

[149] Paszkowski P., Noyce R.S., Evans D.H. (2016). Live-Cell Imaging of Vaccinia Virus Recombination. PLoS Pathog..

[150] Nitsche A., Kurth A., Pauli G. (2007). Viremia in human Cowpox virus infection. J. Clin. Virol..

[151] Sutter G., Wyatt L.S., Foley P.L., Bennink J.R., Moss B. (1994). A recombinant vector derived from the host range-restricted and highly attenuated MVA strain of vaccinia virus stimulates protective immunity in mice to influenza virus. Vaccine.

[152] Lee S.W., Markham P.F., Coppo M.J., Legione A.R., Markham J.F., Noormohammadi A.H., Browning G.F., Ficorilli N., Hartley C.A., Devlin J.M. (2012). Attenuated vaccines can recombine to form virulent field viruses. Science.

[153] Ramirez J.C., Gherardi M.M., Esteban M. (2000). Biology of attenuated modified vaccinia virus Ankara recombinant vector in mice: Virus fate and activation of B- and T-cell immune responses in comparison with the Western Reserve strain and advantages as a vaccine. J. Virol..

[154] Ramirez J.C., Gherardi M.M., Rodriguez D., Esteban M. (2000). Attenuated modified vaccinia virus Ankara can be used as an immunizing agent under conditions of preexisting immunity to the vector. J. Virol..

[155] Stittelaar K.J., Kuiken T., de Swart R.L., van Amerongen G., Vos H.W., Niesters H.G., van Schalkwijk P., van der Kwast T., Wyatt L.S., Moss B. (2001). Safety of modified vaccinia virus Ankara (MVA) in immune-suppressed macaques. Vaccine.

[156] Stittelaar K.J., Osterhaus A.D. (2001). MVA: A cuckoo in the vaccine nest?. Vaccine.

[157] Van den Akker E., van der Vlugt C.J., Bleijs D.A., Bergmans H.E. (2013). Environmental risk assessment of replication competent viral vectors applied in clinical trials: Potential effects of inserted sequences. Curr. Gene Ther..

[158] Sparkes J.D., Fenje P. (1972). The effect of residual moisture in lyophilized smallpox vaccine on its stability at different temperatures. Bull. World Health Organ..

[159] Rheinbaben F.G.J., Exner M., Schmidt M., Mercer A.A., Schmidt A., Weber O. (2007). Environmental Resistance Disinfection and Sterilization of Poxviruses.

[160] Gallina L., Scagliarini A. (2010). Virucidal efficacy of common disinfectants against orf virus. Vet. Rec..

[161] De Oliveira T.M., Rehfeld I.S., Coelho Guedes M.I., Ferreira J.M., Kroon E.G., Lobato Z.I. (2011). Susceptibility of Vaccinia virus to chemical disinfectants. Am. J. Trop. Med. Hyg..

[162] Butcher W., Ulaeto D. (2005). Contact inactivation of orthopoxviruses by household disinfectants. J. Appl. Microbiol..

[163] England L.S., Holmes S.B., Trevors J.T. (1998). Persistence of viruses and DNA in soil. World J. Microb. Biot..

[164] Woo H.J., Reifman J. (2012). A quantitative quasispecies theory-based model of virus escape mutation under immune selection. Proc. Natl. Acad. Sci. USA.

[165] Thomas S., Redfern J.B., Lidbury B.A., Mahalingam S. (2006). Antibody-dependent enhancement and vaccine development. Expert Rev. Vaccin..

[166] Johnson T.R., Graham B.S. (1999). Secreted respiratory syncytial virus G glycoprotein induces interleukin-5 (IL-5), IL-13 and eosinophilia by an IL-4-independent mechanism. J. Virol..

[167] Wyatt L.S., Earl P.L., Eller L.A., Moss B. (2004). Highly attenuated smallpox vaccine protects mice with and without immune deficiencies against pathogenic vaccinia virus challenge. Proc. Natl. Acad. Sci. USA.

[168] Price P.J., Torres-Dominguez L.E., Brandmuller C., Sutter G., Lehmann M.H. (2013). Modified Vaccinia virus Ankara: Innate immune activation and induction of cellular signalling. Vaccine.

[169] Smith G.L., Benfield C.T., Maluquer de Motes C., Mazzon M., Ember S.W., Ferguson B.J., Sumner R.P. (2013). Vaccinia virus immune evasion: Mechanisms, virulence and immunogenicity. J. Gen. Virol..

[170] Clark R.H., Kenyon J.C., Bartlett N.W., Tscharke D.C., Smith G.L. (2006). Deletion of gene A41L enhances vaccinia virus immunogenicity and vaccine efficacy. J. Gen. Virol..

[171] Staib C., Kisling S., Erfle V., Sutter G. (2005). Inactivation of the viral interleukin 1beta receptor improves CD8+ T-cell memory responses elicited upon immunization with modified vaccinia virus Ankara. J. Gen. Virol..

[172] Garcia-Arriaza J., Arnaez P., Gomez C.E., Sorzano C.O., Esteban M. (2013). Improving Adaptive and Memory Immune Responses of an HIV/AIDS Vaccine Candidate MVA-B by Deletion of Vaccinia Virus Genes (C6L and K7R) Blocking Interferon Signaling Pathways. PLoS ONE.

[173] Garcia-Arriaza J., Gomez C.E., Sorzano C.O., Esteban M. (2014). Deletion of the vaccinia virus N2L gene encoding an inhibitor of IRF3 improves the immunogenicity of modified vaccinia virus Ankara expressing HIV-1 antigens. J. Virol..

[174] Chavan R., Marfatia K.A., An I.C., Garber D.A., Feinberg M.B. (2006). Expression of CCL20 and granulocyte-macrophage colony-stimulating factor, but not Flt3-L, from modified vaccinia virus ankara enhances antiviral cellular and humoral immune responses. J. Virol..

[175] Kannanganat S., Wyatt L.S., Gangadhara S., Chamcha V., Chea L.S., Kozlowski P.A., LaBranche C.C., Chennareddi L., Lawson B., Reddy P.B. (2016). High Doses of GM-CSF Inhibit Antibody Responses in Rectal Secretions and Diminish Modified Vaccinia Ankara/Simian Immunodeficiency Virus Vaccine Protection in TRIM5α-Restrictive Macaques. J. Immunol..

[176] Backes S., Sperling K.M., Zwilling J., Gasteiger G., Ludwig H., Kremmer E., Schwantes A., Staib C., Sutter G. (2010). Viral host-range factor C7 or K1 is essential for modified vaccinia virus Ankara late gene expression in human and murine cells, irrespective of their capacity to inhibit protein kinase R-mediated phosphorylation of eukaryotic translation initiation factor 2α. J. Gen. Virol..

[177] Rojas J.J., Sampath P., Bonilla B., Ashley A., Hou W., Byrd D., Thorne S.H. (2016). Manipulating TLR Signaling Increases the Anti-tumor T Cell Response Induced by Viral Cancer Therapies. Cell Rep..

[178] Berger A. (2000). Th1 and Th2 responses: What are they?. BMJ.

[179] Raphael I., Nalawade S., Eagar T.N., Forsthuber T.G. (2015). T cell subsets and their signature cytokines in autoimmune and inflammatory diseases. Cytokine.

[180] Holgado M.P., Falivene J., Maeto C., Amigo M., Pascutti M.F., Vecchione M.B., Bruttomesso A., Calamante G., del Medico-Zajac M.P., Gherardi M.M. (2016). Deletion of A44L, A46R and C12L Vaccinia Virus Genes from the MVA Genome Improved the Vector Immunogenicity by Modifying the Innate Immune Response Generating Enhanced and Optimized Specific T-Cell Responses. Viruses.

[181] Cottingham M.G., Andersen R.F., Spencer A.J., Saurya S., Furze J., Hill A.V., Gilbert S.C. (2008). Recombination-mediated genetic engineering of a bacterial artificial chromosome clone of modified vaccinia virus Ankara (MVA). PLoS ONE.

[182] Perdiguero B., Gomez C.E., Najera J.L., Sorzano C.O., Delaloye J., Gonzalez-Sanz R., Jimenez V., Roger T., Calandra T., Pantaleo G. (2012). Deletion of the viral anti-apoptotic gene F1L in the HIV/AIDS vaccine candidate MVA-C enhances immune responses against HIV-1 antigens. PLoS ONE.

[183] Jackson R.J., Ramsay A.J., Christensen C.D., Beaton S., Hall D.F., Ramshaw I.A. (2001). Expression of mouse interleukin-4 by a recombinant ectromelia virus suppresses cytolytic lymphocyte responses and overcomes genetic resistance to mousepox. J. Virol..

[184] Stanford M.M., McFadden G. (2005). The supervirus? Lessons from IL-4-expressing poxviruses. Trends Immunol..

[185] Bonjardim C.A. (2017). Viral exploitation of the MEK/ERK pathway—A tale of vaccinia virus and other viruses. Virology.

[186] Soares J.A., Leite F.G., Andrade L.G., Torres A.A., de Sousa L.P., Barcelos L.S., Teixeira M.M., Ferreira P.C., Kroon E.G., Souto-Padron T. (2009). Activation of the PI3K/Akt pathway early during vaccinia and cowpox virus infections is required for both host survival and viral replication. J. Virol..

[187] Seo G.J., Yang A., Tan B., Kim S., Liang Q., Choi Y., Yuan W., Feng P., Park H.S., Jung J.U. (2015). Akt Kinase-Mediated Checkpoint of cGAS DNA Sensing Pathway. Cell Rep..

[188] Schweneker M., Lukassen S., Spath M., Wolferstatter M., Babel E., Brinkmann K., Wielert U., Chaplin P., Suter M., Hausmann J. (2012). The vaccinia virus O1 protein is required for sustained activation of extracellular signal-regulated kinase 1/2 and promotes viral virulence. J. Virol..

[189] Pereira A.C., Leite F.G., Brasil B.S., Soares-Martins J.A., Torres A.A., Pimenta P.F., Souto-Padron T., Traktman P., Ferreira P.C., Kroon E.G. (2012). A vaccinia virus-driven interplay between the MKK4/7-JNK1/2 pathway and cytoskeleton reorganization. J. Virol..

[190] Hu W., Hofstetter W., Guo W., Li H., Pataer A., Peng H.H., Guo Z.S., Bartlett D.L., Lin A., Swisher S.G. (2008). JNK-deficiency enhanced oncolytic vaccinia virus replication and blocked activation of double-stranded RNA-dependent protein kinase. Cancer Gene Ther..

[191] Gedey R., Jin X.L., Hinthong O., Shisler J.L. (2006). Poxviral regulation of the host NF-κB response: The vaccinia virus M2L protein inhibits induction of NF-κB activation via an ERK2 pathway in virus-infected human embryonic kidney cells. J. Virol..

[192] Caceres A., Perdiguero B., Gomez C.E., Cepeda M.V., Caelles C., Sorzano C.O., Esteban M. (2013). Involvement of the cellular phosphatase DUSP1 in vaccinia virus infection. PLoS Pathog..

[193] Knol A.B., Petersen A.C., van der Sluijs J.P., Lebret E. (2009). Dealing with uncertainties in environmental burden of disease assessment. Environ. Health.

[194] Skinner D.J.C., Rocks S.A., Pollard S.J.T. (2017). Where do uncertainties reside within environmental risk assessments? Testing UnISERA, a guide for uncertainty assessment. Environ. Pollut..

[195] Wynne B. (1992). Uncertainty and Environmental Learning—Reconceiving Science and Policy in the Preventive Paradigm. Glob. Environ. Chang..

[196] Liu B.F., Bartz L., Duke N. (2016). Communicating crisis uncertainty: A review of the knowledge gaps. Public Relat. Rev..

[197] Skinner D.J.C., Rocks S.A., Pollard S.J.T., Drew G.H. (2014). Identifying Uncertainty in Environmental Risk Assessments: The Development of a Novel Typology and Its Implications for Risk Characterization. Hum. Ecol. Risk Assess..

[198] Skinner D.J.C., Rocks S.A., Pollard S.J.T. (2014). A review of uncertainty in environmental risk: Characterising potential natures, locations and levels. J. Risk Res..

[199] Mhyr A.I., Traavik T., Barrera-Saldana H.A. (2012). Genetically enginneered virus-vectored vaccines—Environmental risk assessment and management challenges. Genetic Engineering—Basics, New Applications and Responsibilities.

[200] Wolt J.D., Keese P., Raybould A., Fitzpatrick J.W., Burachik M., Gray A., Olin S.S., Schiemann J., Sears M., Wu F. (2010). Problem formulation in the environmental risk assessment for genetically modified plants. Transgenic Res..

